# Age-dependent shift in the de novo proteome accompanies pathogenesis in an Alzheimer’s disease mouse model

**DOI:** 10.1038/s42003-021-02324-6

**Published:** 2021-06-30

**Authors:** Megan K. Elder, Hediye Erdjument-Bromage, Mauricio M. Oliveira, Maggie Mamcarz, Thomas A. Neubert, Eric Klann

**Affiliations:** 1 Center for Neural Science, New York University, New York, NY USA; 2 Department of Cell Biology, New York University Grossman School of Medicine, New York, NY USA; 3 Kimmel Center for Biology and Medicine at the Skirball Institute, New York University Grossman School of Medicine, New York, NY USA; 4 NYU Neuroscience Institute, New York University Grossman School of Medicine, New York, NY USA

**Keywords:** Alzheimer's disease, Proteome informatics

## Abstract

Alzheimer’s disease (AD) is an age-related neurodegenerative disorder associated with memory loss, but the AD-associated neuropathological changes begin years before memory impairments. Investigation of the early molecular abnormalities in AD might offer innovative opportunities to target memory impairment prior to onset. Decreased protein synthesis plays a fundamental role in AD, yet the consequences of this dysregulation for cellular function remain unknown. We hypothesize that alterations in the de novo proteome drive early metabolic alterations in the hippocampus that persist throughout AD progression. Using a combinatorial amino acid tagging approach to selectively label and enrich newly synthesized proteins, we found that the de novo proteome is disturbed in young APP/PS1 mice prior to symptom onset, affecting the synthesis of multiple components of the synaptic, lysosomal, and mitochondrial pathways. Furthermore, the synthesis of large clusters of ribosomal subunits were affected throughout development. Our data suggest that large-scale changes in protein synthesis could underlie cellular dysfunction in AD.

## Abstract (web summary)

Elder et al. investigated the de novo proteome in isolated hippocampal slices of young APP/PS1 Alzheimer’s model mice and revealed that large-scale changes in protein synthesis precede symptom onset. These results suggest that broad dysregulation of protein synthesis could contribute toward cellular dysfunction in Alzheimer’s disease.

## Introduction

Alzheimer’s disease (AD) is an age-related neurodegenerative condition characterized by progressive and devastating cognitive impairment. AD is classically characterized by extensive deposition of amyloid beta (Aβ) plaques and intracellular inclusions of hyperphosphorylated tau in the form of neurofibrillary tangles^1^. In addition to the deposition of plaques and tangles, AD is also characterized by extensive atrophy of the brain, which follows a prescribed trajectory throughout disease progression. Initially atrophy is restricted to the hippocampi and medial temporal lobes, even before the appearance of symptoms^2^. The cause of this neuronal atrophy has not yet been elucidated, but it is likely that pathological alterations in protein synthesis are a contributing factor^3^. In a healthy biological system, proteins are made and degraded in a controlled process termed protein homeostasis (proteostasis). However, the intrinsic and extrinsic stressors that accumulate with age and disease can challenge the delicate balance between protein synthesis and degradation^4^. Removal of misfolded or otherwise damaged proteins is essential to avoid toxicity and is regulated by a variety of proteolytic systems whose dysfunction has been implicated in AD pathophysiology^5^. Proteasomal activity is perturbed in vulnerable cortical areas and the hippocampus in late stage AD^6^, whereas proteolysis has been shown to be impaired in very mild stages of the disease suggesting that proteostasis disruption might occur prior to the development of symptoms^7^.

On the opposite side of the proteostatic balance is protein synthesis. Following transcription, mRNA is translated to a polypeptide chain via ribosomal activity, a process that in neurons occurs both somatically and locally in projections^8^. This de novo protein synthesis is a dynamic process that is necessary for basal neuronal function, responds to stimuli that induced long-lasting plasticity and is required for memory consolidation^9,10^. In AD, multiple studies have observed dysregulation of translation throughout the disease process, as indicated by changes in p70 S6 kinase 1, eukaryotic initiation factors 2 alpha (eIF2α) and 4E (eIF4E) phosphorylation^11–16^, as well as decreased ribosomal function^3,17–19^. Curiously, altered expression of ribosomal RNAs and ribosomal protein-coding mRNAs in the hippocampus appear even prior to the onset of symptoms and the development of hallmark AD pathologies^3,17^. Furthermore, ribosomal dysfunction is specific to cortical areas that show the greatest atrophy in later stages of the disease, including the inferior parietal lobule and superior middle temporal gyri^17^.

Although alterations in the level of de novo protein synthesis in AD have been observed in multiple studies, the mechanistic details involved in this dysregulation remain elusive^20^. When monitored in APP/PS1 mutant mice, which express familial AD-associated mutations in the amyloid precursor protein (APP_swe_) and presenilin-1 genes (PS1_ΔE9_)^15^, a ~30% decrease in de novo protein synthesis was observed in aged, symptomatic APP/PS1 mice compared to wild-type littermates^15^. Elucidating the identity of these dysregulated proteins has become an important focus of research, and two independent studies of transgenic mice that experimentally model different aspects of AD pathology have used in vivo metabolic labelling to isolate de novo synthesized proteins for subsequent mass spectrometry analysis^21,22^. The use of a less complex system, such as isolated hippocampal slices, offers a different resolution and a narrower timeframe for this snapshot of the de novo proteome.

Herein, we used BONLAC, a combinatorial approach of stable isotope labelling by amino acid tagging (SILAC) and biorthogonal noncanonical amino acid tagging (BONCAT), and mass spectrometry to compare de novo protein synthesis in acute hippocampal slices from 4-month-old and 12-month-old APP/PS1 mutant mice for the identification and measurement of relative abundance of de novo synthesized proteins^21,23–28^. Here, we show for the first time a substantial dysregulation of proteostasis even before symptom onset in the 4-month-old mice, with alterations in networks of proteins involved in both protein degradation and synthesis. Further, in aged APP/PS1 mice that display memory impairments^29^, we observed dysregulation of both lysosomal and mitochondrial proteins as well as components of the ribosome. Together, these findings suggest that alterations in the synthesis of proteins involved in a variety of critical cellular pathways are impaired early in the AD process and likely underlie the deterioration of neuronal function, leading to loss of memory.

## Results

### Aging-related downregulation of hippocampal protein synthesis in APP/PS1 mutant mice

**Fig. 1:**
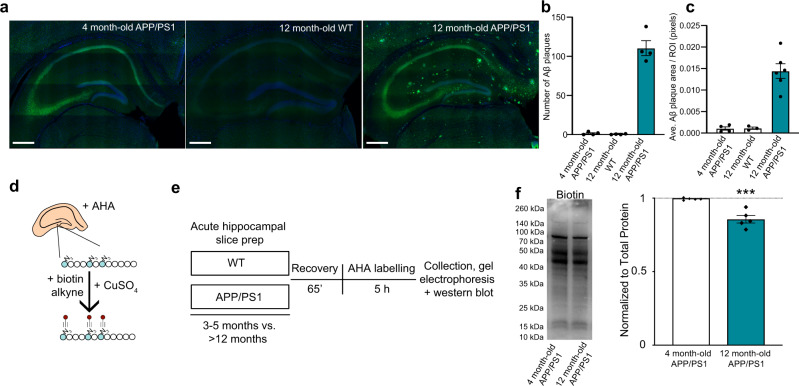
BONLAC-mediated labelling of de novo protein synthesis in the hippocampus of young and aged wild-type and APP/PS1 mice. **a** Representative immunofluorescent images showing the progressive deposition of amyloid beta plaques in the hippocampus of 12-month-old APP/PS1 mice, but not 3–5-month-old APP/PS1 or 12-month-old wild-type (WT) mice (Blue = DAPI, Green = Aβ; scale bar for each micrograph = 400 μm). **b** Quantification of the number of Aβ plaques in the hippocampus of 3–5-month-old APP/PS1 mice, 12-month-old WT and 12-month-old APP/PS1 mice. **c** Quantification of the average area of the Aβ plaques/hippocampus observed in these groups (*n* = 4 biologically independent animals per group for **b** and *n* = 4 biologically independent animals for the 4-month-old APP/PS1 and 12-month-old WT groups, and *n* = 5 biologically independent animals for the 12-month-old APP/PS1 animals. **c**, **d** Schematic showing BONCAT labelling technique for acute hippocampal slices. **e** Workflow for processing of BONCAT-labelled tissue from WT and APP/PS1 mice. Following labelling, slices are processed for western blot detection of AHA incorporation. **f** *De novo* synthesized protein in 3–5-month-old vs. 12+-month-old APP/PS1 mouse hippocampal slices as detected via AHA labelling, followed by biotin-alkyne click reaction and western blot (normalized to total protein (as determined via MemCode staining), expressed relative to average 3–5-month-old biotin signal; *n* = 5 biologically independent animals per group; statistical significance calculated using unpaired two-tailed *t*-test: *t* = 5.545, 95% confidence interval = −0.02033 to −0.08388, effect size = −0.1436 ± 0.0259, *df* = 8, *p* = 0.0005); graphs in **b**, **c** and **f** show mean ± SEM.

Previous studies using puromycin and azidohomoalanine (AHA) incorporation to label newly synthesized proteins in mice modeling aspects of AD pathology demonstrated a reduction in de novo protein synthesis^15,21,22,30,31^, and the translation efficiency of polyribosomes isolated from the mild cognitive impairment (MCI) and end-stage AD brain is reduced by >60% in affected cortical areas^17^. We first confirmed that histopathological changes are observed in the brain of 4 vs 12-month-old APP/PS1 mice by performing immunohistochemistry against Aβ in brain sections including the dorsal hippocampus. As expected, we found that the amyloid deposits are severely increased in the brains of aged APP/PS1 mice when compared to younger APP/PS1 mice (Fig. 1a–c, Supplementary Data 1). Using BONCAT labeling (Fig. 1d, e), we observed a ~20% decrease in the level of de novo protein synthesis in 12-month-old APP/PS1 mice compared to 3–5-month-old APP/PS1 mice (Fig. 1f, Supplementary Data 1). Collectively, these findings support and extend previous work conducted in our laboratory, which highlighted a significant decrease in de novo protein synthesis between aged WT and APP/PS1 mice using SUnSET, another non-radioactive method for tagging and monitoring new protein synthesis^15^. Therefore, using BONLAC labeling we proceeded to identify changes in the de novo proteome in the young and aged APP/PS1 mutant mice.

### Large-scale proteomic dysfunction is observed early in the hippocampus of APP/PS1 mice

**Fig. 2:**
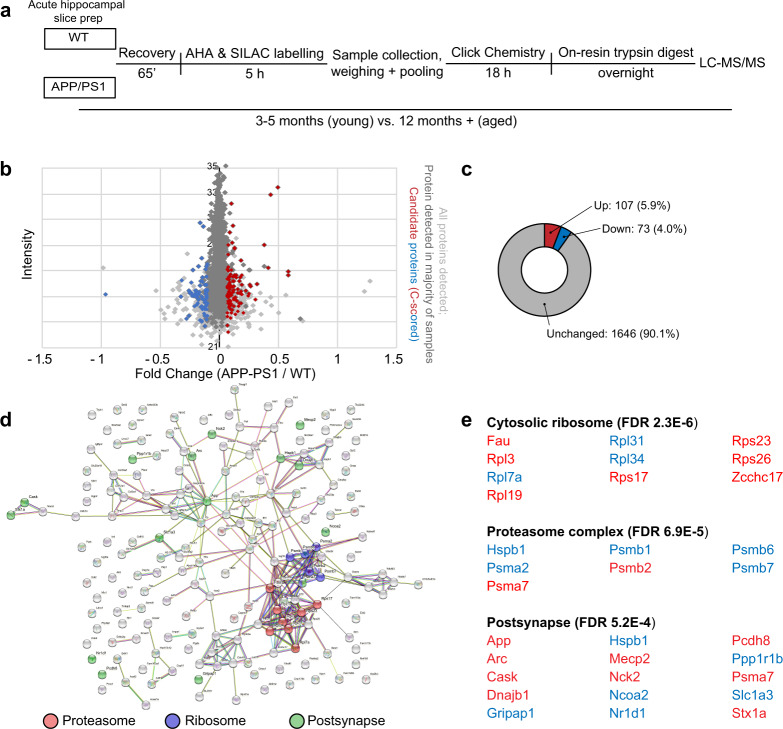
Steady state proteome differs in young wild-type and APP/PS1 mouse hippocampus. **a** Schematic and timeline of BONLAC labelling protocol. **b** Fold change vs. intensity distribution plot of all newly made proteins detected in wild-type (WT) vs. 3–5-month-old APP/PS1 hippocampus (light grey), with proteins that were detected in majority of samples (>3 out of five biologically independent samples; grey) and dysregulated candidate proteins identified by C-score screen (upregulated ≥ 20% in red, downregulated ≤ 20% in blue). **c** Doughnut plot showing the proportion of detected proteins that were downregulated (fold change < 0.8, blue), upregulated (fold change > 1.2, red) or unchanged in the majority of 3–5-month-old APP/PS1 mice compared to WT littermates. **d** String diagram depicting enriched gene ontology groups. **e** Top functional clusters in 3–5-month-old APP/PS1 mice compared to WT using DAVID. Red = upregulated proteins. Blue = downregulated proteins.

In order to identify candidate proteins of interest, we used BONLAC and a previously published multi-layered analysis based in coincidence detection^23,28^. In the hippocampi of 3–5-month-old mice, 2510 de novo synthesized proteins were measured in at least one sample. One thousand eight hundred twenty six proteins were measured across the majority of samples (≥3/5 samples; Supplementary Fig. 1), and of these 1826, 180 were differentially regulated in the APP/PS1 mice (≥20% fold change; Fig. 2a; Supplementary Fig. 2, Supplementary Data 1). The majority of differentially regulated proteins were downregulated (fold change ≤ 0.8; 107 or 5.9% of total proteins), whereas 73 proteins were upregulated (fold change ≥ 1.2; 4.0% of total proteins; Fig. 2b, Supplementary Data 1) in the APP/PS1 mice. When the biological relevance of these changes was interrogated using DAVID and StringDB, the GO and KEGG pathways that were highlighted included the cellular component ‘postsynapse’, as well as proteostasis-related components, gathered under the terms ‘proteasome’ and ‘ribosome’ (Fig. 2c, d, Supplementary Data 1). These findings indicate that even in young APP/PS1 mice that do not show memory deficits, processes that are both previously linked to AD and are critical to cell functioning are disturbed.

### Alterations in the de novo proteome correspond with pathologies in aged APP/PS1 mice

**Fig. 3:**
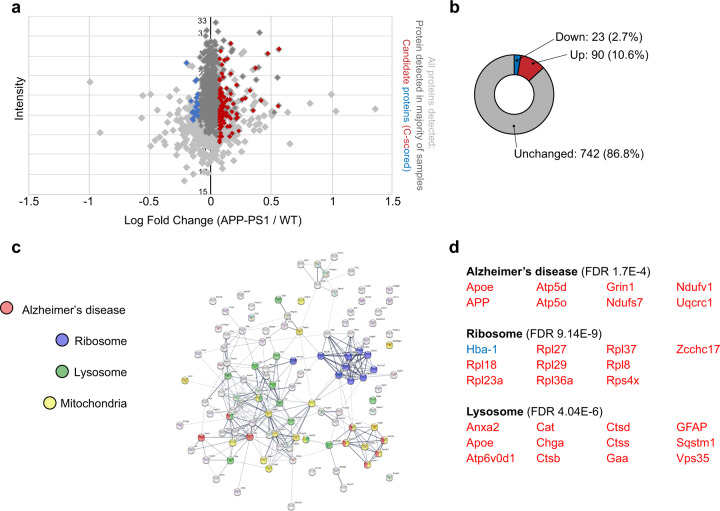
Analysis of altered de novo proteome highlights impaired proteostasis in aged APP/PS1 mice. **a** Fold change vs. intensity distribution plot showing all proteins detected in 12+-month-old APP/PS1 mouse hippocampal slices vs. wild-type (WT) littermates using BONLAC (light grey). Dark grey overlay depicts proteins that were consistently detected in the majority of samples (>4 out of 7 biologically independent samples), and candidate proteins identified by C-score screen as upregulated (fold change ≥ 1.2) shown in red, while downregulated proteins (≤0.8) are labelled blue. **b** Doughnut plot indicating the majority of proteins detected in 12+-month-old mice are not altered in APP/PS1 mice (742; 86.8% of proteins detected). 23 proteins are downregulated compared to WT mice (2.7% of proteins show a fold change > 0.8, blue) and 90 proteins are upregulated in APP/PS1 mice (10.6% of proteins show a fold change < 1.2, red). **c** String diagram showing enriched gene ontology networks. **d** DAVID-identified functional clusters in 12+-month-old APP/PS1 mice. Red = upregulated proteins. Blue = downregulated proteins.

Following these investigations in young APP/PS1 mice, we investigated the impact of aging and the development of AD-like pathology on the de novo proteome. As expected from the decreased de novo translation apparent in AHA incorporation levels (Fig. 1f), fewer newly synthesized proteins were observed in ≥12-month-old APP/PS1 mice compared to younger animals. In the hippocampi of aged mice, 2065 proteins were measured at least once (Supplementary Fig. 3), 855 proteins were measured across the majority of samples (≥4/7 samples) and 113 proteins were consistently altered in the APP/PS1 mice (≥20% fold change; Fig. 3a; Supplementary Fig. 4, Supplementary Data 1). Proteins whose synthesis was reduced by 20% compared to age-matched WT mice made up 2.7% of the consistently detected proteins (23 proteins), while BONLAC detected 90 proteins with increased de novo synthesis in the APP/PS1 mice (10.6%; Fig. 3b, Supplementary Data 1). Through GO analysis via DAVID and visualization using StringDB, we observed several functional clusters (Fig. 3c, Supplementary Data 1), with the top hits including ‘Alzheimer’s disease’, ‘ribosome’ and ‘lysosome’ (Fig. 3d). Together, these findings indicate that the dysregulation of core cellular processes relies at least partially on impaired protein synthesis, and that changes in de novo proteome happen even before full pathology onset.

### Dysregulation of the synthesis of BONLAC-identified candidates is reflected in protein expression

**Fig. 4:**
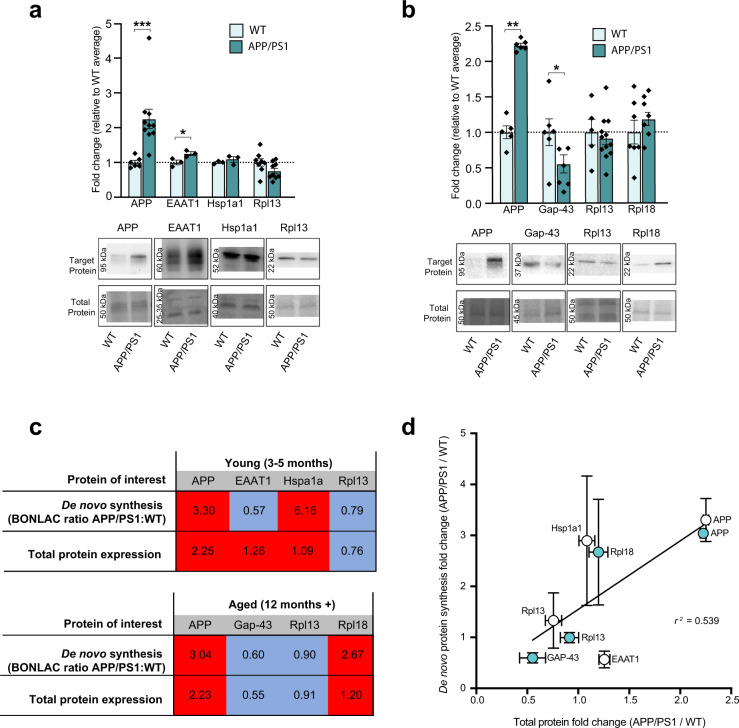
Candidate proteins identified with BONLAC screen of de novo proteome exhibit altered expression levels in APP/PS1 mice. **a** Western blot quantitation of candidate proteins selected from BONLAC screen in 3–5-month-old wild-type (WT; light bars) and APP/PS1 (dark bars) mice, normalized in- lane to total protein (as assessed by MemCode or Ponceau stain [note only a portion of the entire analyzed lane is presented in the figure]) and expressed relative to age-matched WT (Number of biologically independent animals samples: APP 3–5 month *n* = 8, 12+ month *n* = 6; EAAT1 *n* = 3; Gap-43 *n* = 6; Hsp1a1 *n* = 3; Rpl13 3–5 month *n* = 9, 12+ month *n* = 12; Rpl18: *n* = 7); representative western blots show selected protein levels in WT and APP/PS1 mouse hippocampal lysates. **b** Representative western blot and quantitation of candidate proteins selected from BONLAC screen in 12+-month-old WT and APP/PS1 mice. **c** Comparison table showing average fold change in de novo synthesis of candidate proteins in young and aged APP/PS1 vs WT littermates as identified by BONLAC (young *n* = 5 biologically independent samples; aged *n* = 7 biologically independent samples) compared to change in total expression quantified by western blot. Statistical significance calculated between APP/PS1 and WT samples using unpaired two-tailed *t*-tests; young APP: *t* = 3.378, 95% confidence interval = 0.4571–2.047, effect size = 1.252 ± 0.3707, *df* = 14, *p* = 0.0045; young EAAT1: *t* = 2.945, 95% confidence interval = 0.0146–0.4966, effect size = 0.2556 ± 0.0868, *df* = 4, *p* = 0.0422; aged APP: *t* = 13.95, 95% confidence interval = 1.027–1.424, effect size = 1.226 ± 0.08782, *df* = 9, *p* < 0.0001; aged GAP-43: *t* = 2.266, 95% confidence interval = −0.9047 to −0.01774, effect size = −0.4612 ± 0.2035, *df* = 12, *p* = 0.0427. **d** Linear regression showing correlation between average de novo synthesized protein fold change (as determined by BONLAC) vs. average total protein fold change (as determined via western blot) of candidate proteins in the APP/PS1 vs. WT hippocampus at 3–5 months (white circles) and 12+ months of age (teal circles; *r* ^*2*^ value = 0.690, vertical error bars: BONLAC; horizontal error bars: western blot; *p* = 0.0207). Graphs in **a**, **b** and **d** show mean ± SEM.

Next, to understand whether these changes in de novo protein synthesis corresponded to altered protein expression, we validated candidates from several enriched clusters using western blotting, comparing changes in expression between APP/PS1 and wild-type littermates. Alongside APP, which was selected as a positive control^32^, other AD-associated proteins known to be involved in regulating glial proteostasis (heat shock 70 kDa protein 1 A; hspa1a/hsp68^33^) and synaptic plasticity (neuromodulin; GAP-43^34,35^) were probed. Samples were also probed for the expression of the large 60 S ribosomal proteins Rpl13 and Rpl18. As shown in Fig. 4a–c (and Supplementary Data 1), both the de novo synthesis and the total expression of APP were increased in young and aged APP/PS1 mice, as expected. The synthesis and expression of Hsp1a1 protein were altered in young APP/PS1 mice compared to wild-type littermates. However, although the synthesis of the excitatory amino acid transporter EAAT1 was decreased in young APP/PS1 mice compared to WT, total protein expression was elevated. In aged APP/PS1 mice, a decrease in both the synthesis and total expression of the synaptic protein GAP-43, which corresponds with a known decline in synaptic density at this age^36^.

The large 60S ribosomal subunit protein Rpl13 also was profiled after BONLAC labelling indicated hippocampal synthesis of this protein was decreased in APP/PS1 mutant mice throughout aging. Western blot analysis confirmed that there was a reduction in expression of this ribosomal protein in both groups of APP/PS1 mice. The expression of an additional ribosomal protein, Rpl18, was investigated as BONLAC labelling indicated elevated synthesis of this protein in aged APP/PS1 mice. Both newly synthesized and total expression of Rpl18 were increased at trend levels relative to wild-type mice, although no significance was observed.

Next, we performed a linear regression analysis between the BONLAC and western blot fold changes (APP/PS1 vs. WT) to evaluate whether a linearity existed between the values. Linear regression analysis highlighted a positive correlation between detection of the candidate protein in the de novo analysis vs. candidate protein expression in the total protein fraction (Fig. 4d, Supplementary Data 1). Together, these results indicate that altered synthesis of proteins detected with BONLAC are correlated with changes in global protein expression.

### Cluster analysis of commonly synthesized proteins in both young and aged APP/PS1 mice highlights key AD pathways

**Fig. 5:**
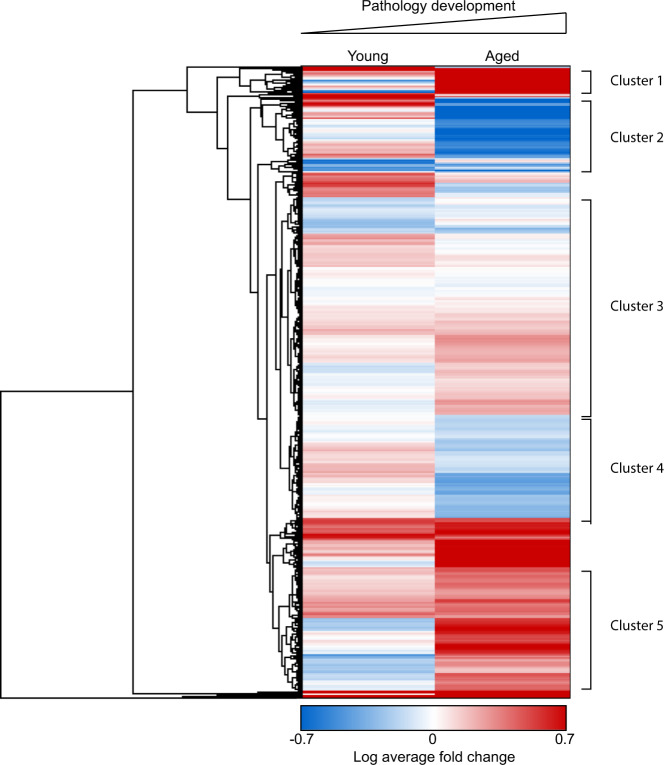
The biological pathways affected by dysregulated hippocampal protein synthesis vary with age in APP/PS1 mice. Hierarchical clustering heatmap reveals similarities and divergence in the mean-normalized log protein fold change of the 791 proteins detected in both young (3–5 months old) and aged (12+ months old) APP/PS1 mice relative to wild-type (WT) littermates. GO analysis evidenced six major clusters being modified, which are: (1) protein processing in the endoplasmic reticulum (FDR 3.2E-04); (2) synapse (FDR 5.6E-07); (3) synaptic vesicle cycle (FDR 2.77E-06), membrane trafficking (FDR 6.24E-11) and synapse (FDR 4.82E-29); (4) axo-dendritic transport (FDR 4.86E-08) and glycolysis/gluconeogenesis (2.56E-05); (5) myelin sheath (5.17E-18) and Alzheimer’s disease (8.16E-07); and (6) mitochondrial part (FDR 2.54E-08) and electron transfer activity (FDR 4.02E-05). FDR generated by StringDB algorithms. Red = upregulated proteins. Blue = downregulated proteins. White = unchanged protein expression.

Following the corroboration of BONLAC candidates by western blotting, we further investigated the overall patterns of protein synthesis regulation observed in the APP/PS1 mice. Although examination of changes detected by this stringent analysis reveal statistically relevant alteration of the de novo proteome, it is noteworthy that smaller fluctuations in expression (fold change ≤ 20%: labelling ratio ≥ 0.8 or ≤1.2) can also provide information as to whether a specific cell process is altered. Therefore, we compared all 791 newly synthesized proteins identified in both 3–5-month-old and 12+-month-old APP/PS1 mice and their wild-type littermates and identified any candidates that were up- or downregulated by ≤15% (labelling ratio ≥0.85 or ≤1.15*)*. To investigate whether any biological pathways were differentially affected, we conducted hierarchical clustering analysis using Cytoscape. Several key clusters were observed (Fig. 5, Supplementary Table 1, Supplementary Data 1), closely associated with the KEGG pathways ‘Alzheimer’s disease’ (Fig. 5 Cluster 5; Supplementary Fig. 5, Supplementary Data 1) and ‘protein processing in the endoplasmic reticulum’ (Fig. 5 Cluster 1; Supplementary Fig. 6, Supplementary Data 1). The GO terms ‘synapse’ (Fig. 5 Cluster 2; Supplementary Fig. 7, Supplementary Data 1) and ‘synaptic vesicle cycle (Fig. 5 Cluster 3; Supplementary Fig. 8, Supplementary Data 1) were enriched in this analysis, as were the GO terms ‘axo-dendritic transport’ (Fig. 5 Cluster 4; Supplementary Fig. 9, Supplementary Data 1), ‘myelin sheath’ (Fig. 5 Cluster 5; Supplementary Fig. 5, Supplementary Data 1), ‘mitochondrial part’ and ‘electron transfer activity’ (Fig. 5 Cluster 6; Supplementary Fig. 10, Supplementary Data 1).

### Dysregulation of ribosomal protein synthesis is a common feature in APP/PS1 mice throughout the aging process

**Fig. 6:**
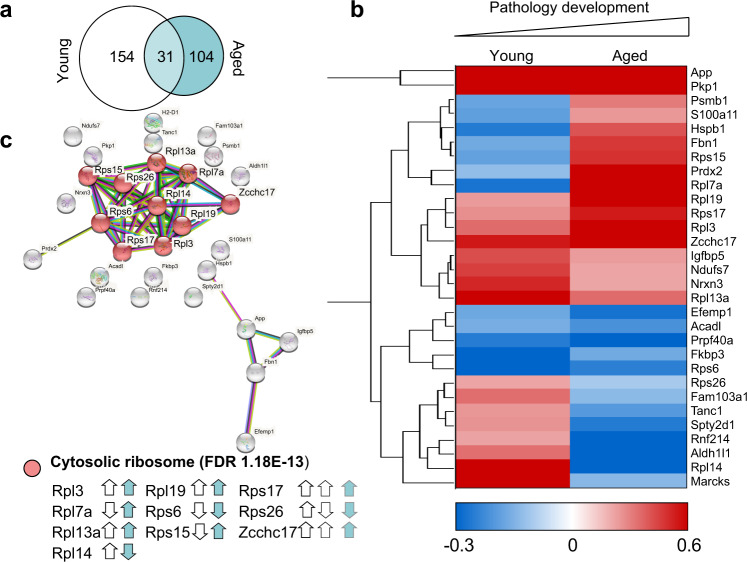
Steady state proteome is predominantly distinct throughout aging. **a** Minimal overlap is observed between proteins that are dysregulated (as detected by C-score rank algorithm) in the hippocampi of 3–5-month-old (young) vs. 12+-month-old (aged) APP/PS1 mice. **b** Hierarchical clustering of log fold changes reveals the majority of proteins, which are dysregulated in both young and aged APP/PS1 mice compared to WT littermates do not show the same trend. Red = upregulated proteins. Blue = downregulated proteins. White = unchanged protein expression. **c** String diagram revealing an enrichment of proteins in the GO network ‘Cytosolic ribosome’ in APP/PS1 throughout the aging process. White arrows = direction of regulation in young mice. Teal arrows = direction of regulation in aged mice.

As described above, C-score detection of candidates generated a list of proteins that were highly dysregulated in the APP/PS1 hippocampus (±20% compared to wild-type). When these dysregulated candidate proteins were compared between age groups, only 31 proteins were dysregulated in both the young and aged APP/PS1 mice relative to age-matched WT littermates (Fig. 6a). The majority of these proteins showed either an increased level of synthesis at both ages (11/31) or reversed expression (decreased synthesis in young APP/PS1 mice and increased synthesis in aged mice, or vice versa; 15/31) in comparison to wild-type mice. Only five proteins showed consistently decreased synthesis in the APP/PS1 mice, regardless of age (Fig. 6b, Supplementary Data 1). Identification of the proteins consistently dysregulated in the APP/PS1 mice highlighted a large cluster of ribosomal proteins, three of which are components of the small 40 S ribosomal subunit, and six of which are components of the large 60 S ribosomal subunit (Fig. 4c). Taken as a whole, these results support the notion that alterations in the expression of ribosomal proteins is an early feature of AD pathology and may well play a role in protein synthesis dysfunction through the course of disease.

## Discussion

Dysregulated proteostasis underlies many of the hallmark pathologies of AD. Both the deposition of Aβ in the form of plaques and accumulation of hyperphosphorylated tau-containing tangles suggest impaired protein degradation systems, and indeed, dysfunctional proteolysis has been observed in AD brains^5^. Further, translation is impaired throughout the AD process^10–17^ and de novo protein synthesis is decreased in mouse lines that model various aspects of AD pathology^15,21,31^. The identity of the proteins, which are differentially synthesized before and after AD symptom onset in the human brain is unknown. Interestingly, however, ribosomal function was shown to be impaired in the brains of individuals diagnosed with mild cognitive impairment^17^.

Recently, the de novo proteome of mouse lines that model aspects of AD have become a focus of study. Decreased de novo protein synthesis was observed in neurons with high levels of hyperphosphorylated tau in the K369I (K3) and rTg4510 transgenic mouse models of tauopathy and neurodegeneration^21^. Moreover, and consistent with our findings, investigation using in vivo metabolic labelling has highlighted significant dysregulation of the synthesis of proteins involved in vesicle transportation and mitochondrial functioning prior to symptom onset in APP/PS1 mice^31^. This study also investigated changes in the proteome both pre- and post-symptom onset, but the young, asymptomatic mice selected for the study (2 months old) do not yet show hallmark Aβ deposition in the hippocampus, which begins at 3–4 months of age^32^. Further, although no memory deficits are observed by this age, alterations in neuronal excitability have previously been described in the 4-month-old APP/PS1 hippocampus^37^. Finally, although significant impairments in hippocampal LTP and cognitive ability are apparent by 9 months of age^32,38,39^, the age selected for the symptomatic group in this work, several studies have indicated that older mice (>12 months old) possess greater deficits in spatial learning and memory^40–43^.

In the present study, we used BONLAC labelling in acute hippocampal slices from 3–5-month-old and ≥12-month-old symptomatic APP/PS1 mice and their wild-type littermates to isolate and identify alterations in the de novo hippocampal proteome. The use of animals at these ages both supports the recent in vivo work described above^31^, and extends the coverage of the de novo proteome investigations throughout pathology development in this model. Decreased synthesis was observed in aged APP/PS1 mice, as represented by lower AHA incorporation and fewer proteins identified in the mass spectrometry screen, as anticipated from prior work indicating an age-dependent decrease in translation in this AD mode^15^. At both timepoints, the vast majority (>85%) of measured proteins were synthesized consistently regardless of genotype, further illustrating the tight regulation of the proteome required for cell functioning. However, defined patterns of dysregulation were observed in APP/PS1 mice relative to unaffected littermates. Indeed, the positive, low-level correlation observed between changes in de novo synthesis and candidate protein expression in the total protein fraction (Fig. 4d) indicates that even in a stressed neural environment overloaded with amyloid beta (as observed in the APP/PS1 mouse model), proteostatic mechanisms still attempt to mitigate the deleterious effects of proteotoxicity^44^.

In young mice without extensive Aβ protein deposition, we observed a specific pattern of dysregulated protein synthesis, resulting in predominately decreased translation of proteins involved in a series of pathways critical to cell functioning. Of note, proteins involved in both protein degradation and protein synthesis showed dysregulated synthesis in the APP/PS1 hippocampus relative to wild-type littermates. In fact, the dysfunctional production of components of the proteasome could have a negative effect on ribosomal function and general protein synthesis, as an interplay between impaired proteasomal function and translation has previously been described^17^. Dysregulated synthesis of synaptic proteins were also highlighted at this time point, which could underlie the decreased synaptic density previously observed in 4-month-old APP/PS1 mice^45^, as well as the altered excitability seen by 4 months^37^, and impaired synaptic transmission observed in APP/PS1 mice at 5 months of age^46^. Thus, our findings may represent a critical window in which modulation of translation limits synaptic changes that result in memory deficits later in disease progression.

When de novo protein synthesis was examined in 12+-month-old APP/PS1 mice that display pronounced AD-like pathology and memory impairment, a large number of mitochondrial proteins previously linked to AD were dysregulated. Mitochondrial structure and function is known to be compromised in human patients^47,48^, but whether these deficits contribute to the disease or whether they are a biproduct of disease progression continues to be debated^49^. Alongside mitochondria, lysosomal protein synthesis was dysregulated in the symptomatic APP/PS1 mice. Increasing evidence suggests that lysosomal biogenesis is initially upregulated in early stages of AD^50^, before progressive dysfunction throughout the disease process results in the accumulation of enlarged lysosomes, which fail to effectively degrade their contents^51^.

A final intriguing pathway that was observed in the analysis of dysregulated proteins following symptom onset was constituents of the cytosolic ribosome, including eight ribosomal proteins and nucleolar protein of 40 kDa (Zcchc17). This zinc ion binding protein has recently been identified as a modulator of synaptic gene expression in AD^52^ and is thought to be involved in ribosome biogenesis and maturation^53^. Two protein components of the ribosome that were differentially regulated in the mature APP/PS1 tissue, Rpl13 (decreased BONLAC labelling compared to wild-type) and Rpl18 (upregulated in APP/PS1 mice) were selected for validation analyses in hippocampal lysates. The total expression of both proteins reflected the level of synthesis as detected in the mass spectrometry screen, indicating that these are biologically relevant changes that require further investigation. Rpl13 has recently been described as a ‘core’ ribosomal protein included in all actively translating ribosomes^54^, and yet has also been shown to be dysregulated at the gene expression level in hippocampal tissue from severe AD patients^55^. Although no study has yet linked alterations in Rpl18 expression with AD pathology, recent analysis has indicated dysregulation of *Rpl18* gene expression occurs early in the AD process^56^.

In order to determine the full impact of APP/PS1 mutations on protein synthesis in these mice, we examined the biological pathways that exhibited changes across pathology development as detected by hierarchical cluster analysis of all proteins detected in both age groups. This list was not limited to proteins identified by C-score analysis as dysregulated, and included proteins detected in the majority of samples, which showed a fold change of <20%. Several functional clusters emerged, indicating pathways specific to stage of pathology development. Two clusters were specifically upregulated in the symptomatic APP/PS1 mice, those associated with the GO terms ‘protein processing in the endoplasmic reticulum (ER)’ and ‘mitochondrial part: electron transfer activity’. Increased synthesis of proteins, including Stub1/CHIP, Cat and Hsph1, which have previously been implicated in the AD process^57–59^, indicate that the unfolded protein response (UPR), the physiological response to the ER stress, might be chronically activated later in disease. This finding is consistent with our previous studies showing that reducing the expression of PERK, an eIF2α kinase that triggers downregulation of protein synthesis in response to UPR activation, could restore hippocampal plasticity and memory deficits in symptomatic APP/PS1 mice^15^.

Mitochondrial dysfunction is known to be an early event in both APP/PS1 mice^60^ and human AD brain pathology^61–63^, and is believed to play a role in the synaptic loss that occurs early in the disease process^64^. Downregulated synthesis of mitochondrial proteins, specifically the oxidative phosphorylation-associated proteins Ndufs5, Ndufa12, Cox4i1 and Cox6b in the younger APP/PS1 mice could underlie the decreased electron transport chain capacity observed in this and other models of AD-like pathology^65,66^.

Functional clusters that exhibited downregulation in the aged APP/PS1 mice compared to the young cohort were also observed. The first cluster was associated with the GO term ‘synapse’, which may be a function of the decreased spine density previously observed in symptomatic APP/PS1 mice^45^. Proteins found in this hub included those involved in glutamatergic signaling, known to be impaired in both the human AD brain and APP/PS1 mice^67^. In addition, a cluster corresponding to ‘glycolysis and gluconeogenesis’ was decreased in the aged APP/PS1 hippocampal de novo proteome. Decreased glucose metabolism has been observed in the human AD brain using radioactive glucose labelling and PET scanning, and correlates well with AD-associated pathologies^68–70^.

Although the cluster analysis described above examined general trends, we also conducted focused analysis using C-score-mediated detection of dramatic changes in de novo protein synthesis, where candidate proteins were selected for further analysis if they showed a fold change of >20% in either direction in APP/PS1 mice vs. wild-type littermates. Comparison of proteins selected in this manner revealed that although few proteins were dysregulated in both age groups, one third of these proteins were identified as ribosomal subunits. The ribosome is composed of four ribosomal RNA molecules and 80 ribosomal proteins^71^, which together form the small and large ribosomal subunits that function together to translate mRNAs into proteins. Recent examination of de novo hippocampal protein synthesis following prolonged in vivo metabolic labelling revealed dysregulated synthesis of Rps3a^31^. Moreover, dysregulated expression of genes encoding several of the ribosomal proteins observed in our study (Rpl7, Rps6, Rps17 and Rps26) has previously been described in the AD brain^3^. Notably, decreased synthesis of ribosomal proteins is a common feature in both amyloid and tauopathy mouse models, as a recent de novo proteomic study using K3 and rTg4510 models of tauopathy revealed dysfunctional translation of these proteins^21^.

The protein content of functional ribosomes was long thought to be largely homogenous, but recent studies have indicated sub-stoichiometric inclusion of ribosomal proteins in actively translating ribosomes in mammalian cells^54,72^. In neurons, active remodeling of ribosomal protein content has been observed in response to axonal stimulation^73^. Notably, ribosomal protein gene expression is regulated throughout the aging process in the brain^74^, indicating that the dynamic regulation of ribosomal components may play a role in cellular adaptation to aging. Further, deficiency in particular ribosomal proteins results in hypersensitivity to deficits in the protein degradation system^75^. Importantly, the protein constituents of the actively translating ribosome appear to confer a level of preference for which mRNAs are translated, suggesting a further level of translational control^54^. Together with the aforementioned studies, our findings make a strong case for further detailed investigations into the dysregulation of ribosomal protein synthesis and its subsequent consequences in AD.

One important consideration when using BONLAC labeling is that the primary output is the ratio of medium to heavy isotope labelling, which is used to generate the fold changes that permit relative quantitation. A caveat to using this system in AD (in which translation is known to be impacted) is that if a protein is not synthesized at all during the labeling window, or synthesis levels are below detection in one sample within the pair, a ratio will not be generated. As we have observed, both here (Fig. 1f) and previously^15,31^, a significant decline in gross de novo protein synthesis in the APP/PS1 mouse^15,31^, this inherent bias may have led to an exaggerated detection of upregulated vs. downregulated proteins in the symptomatic APP/PS1 hippocampal samples. Bioinformatic comparison of the de novo hippocampal proteome generated in this, and recent in vivo analysis^31^, with the basal proteome could reveal proteins that were not synthesized over the labelling period and were thus precluded from BONLAC ratio generation. Further by combining BONLAC labelling and TMT sample multiplexing, the number of missing peptide quantification values in each sample is greatly reduced, providing deeper coverage of the de novo proteome^76^. The incorporation of this approach into subsequent studies promises to reveal insights into the cellular processes that accompany pathology progression in the APP/PS1 mouse.

In summary, we have illustrated a compelling picture of dysregulation of the de novo proteome in the APP/PS1 mouse model of AD-like amyloidy throughout pathology development. Following validation of several candidate proteins to support the relevance of changes observed in the mass spectrometry screen, we conducted robust bioinformatic analysis of the newly made proteins. We observed dysregulated synthesis of proteins involved in several pathways known to be altered throughout the course of AD, including those involved in synaptic, mitochondrial and lysosomal functions, and also observed disturbance of protein components of the ribosome. In light of recent research that indicates inclusion or exclusion of specific ribosomal subunits bestows selectivity to mRNA translation, the up- and downregulation of ribosomal protein synthesis identified here, even prior to the development of pathology, may underly the progressive deterioration of cellular function and memory observed in this AD model, and in individuals with AD.

## Methods

### Animals

All procedures involving animals were performed in accordance with protocols approved by the New York University Animal Welfare Committee and followed the National Institutes of Health (NIH) *Guide for the Care and Use of Laboratory Animals*. All mice were housed in the New York University animal facility. Mice of both sexes were used. APP/PS1 transgenic mice (B6;C3-Tg(APPswe, PSEN1/dE9)85Dbo/Mmjax) and their wild-type littermates were bred and maintained on C57-BL6 and B6.C3 (Jackson Labs) backgrounds. Mice were housed with their littermates in groups of two to three animals per cage and kept on a 12-h regular light/dark cycle, with food and water provided ad libitum. All genotypes were verified by polymerase chain reaction. Mice of both sexes were used at an age of either 3–5 months or 12–15 months.

### Immunohistochemistry

Mice were deeply anesthetized with ketamine (150 mg/kg) and transcardially perfused with 0.1 M PBS followed by 4% PFA before brains were removed and post-fixed for 48 h. Forty-micrometres free-floating coronal sections containing the hippocampus were cut using a Leica vibratome, collected and stored at 4 °C in 0.01% sodium azide until use. Sections were permeabilized in 0.5% Triton X-100 (15 min) prior to blocking (5% normal goat serum, 0.1% Triton X-100; 1 h). Slices were incubated overnight at 4 °C in rabbit anti-amyloid beta antibody (1:200; clone 6E10, ENZ-ABS612-0200, Enzo Life Sciences), followed by Alexa-488-labelled goat anti-rabbit secondary antibody (1:500; RT, 1.5 h). Slices were mounted using ProLong Gold Antifade Mountant with DAPI. Tile-scan z stack images (10–15 optical sections depending on slice thickness) were collected using an SP8 confocal microscope (Leica) with a ×20 magnification lens and Leica LASX software, with laser intensity, smart gain and offset maintained throughout the experiment. Images were processed using ImageJ 2.0.0 using the Bio-Formats importer plug-in. Plaque number was manually quantified using the Cell Counter plug-in (*n* = 4–5 animals/group, 1 dorsal hippocampal slice/animal).

### AHA and SILAC dose

AHA was purchased from Click Chemistry Tools, AZ, USA and SILAC amino acids (13C6-15N2-lysine, 13C6-15N4-arginine (Lys8/Arg10) and D4-lysine/13C6-arginine (Lys4/Arg6)) were obtained from Cambridge Isotope Laboratories, MA, USA and used at previously described concentrations^23,24^. Assignment of AHA and SILAC labels was alternated between biological replicates to ensure that results were not biased by labelling^23^.

### Acute hippocampal slice preparation and incubation

Four hundred micrometres transverse hippocampal slices were obtained from APP/PS1 and WT mice (3–5 months old or 12+ months old of either sex)^77–79^. Mice were cervically dislocated and brains were quickly removed. Transverse hippocampal slices were isolated with a VT1200 Vibratome (Leica) in ice-cold cutting solution (in mM: 110 sucrose, 60 NaCl, 3 KCl, 1.25 NaH_2_PO_4_, 28 NaHCO_3_, 0.5 CaCl_2_, 7 MgCl_2_ and 5 glucose. Slices were recovered and incubated in scintillation tubes with artificial cerebrospinal fluid (ACSF; 125 NaCl, 2.5 KCl, 1.25 NaH_2_PO_4_, 25 NaHCO_3_, 25 glucose, 2 CaCl_2_ and 1 MgSO_4_) for 20 min at room temperature, then at 32 °C for 45 min, constantly bubbled with 95% O_2_/5% CO_2_. AHA (final concentration 1 mM) and SILAC amino acids (final concentrations in mM: 0.798 lysine [^13^C_6_-^15^N_2_-lysine and D_4_-lysine], 0.398 arginine [^13^C_6_–^15^N_4_-arginine and ^13^C_6_–arginine]; Cambridge Isotope Laboratories, MA, USA) were added directly to the ACSF, and slices were incubated for 5 h^23^. Assignment of SILAC labels to experimental conditions (wild-type or APP/PS1) was alternated between biological replicates to ensure that results were not biased by labelling. Following the labelling period, slices were immediately flash frozen for mass spectrometry and stored at −80 °C until use.

### BONLAC sample preparation for mass spectrometry

Samples were prepared using the Click-IT Protein Enrichment Kit (ThermoFisher Scientific)^23^. BONLAC was carried out with a minimum of five runs per condition, with each run examining hippocampal slices from one WT and one age-matched APP/PS1 animal (*n* = 5–7 biological replicates made up of 1 APP/PS1 and 1 WT; 5–7 animals of each age per genotype were used in total). Briefly, following labeling, equal weights of tissue (15 mg each) from age-matched pairs of WT and APP/PS1 animals were lysed together in 850 μL buffer containing 8 M urea, 200 mM Tris pH 8, 4% CHAPS, 1 M NaCl and protease inhibitor cocktail (cOmplete, Mini, Roche; two tablets per 10 mL of lysis buffer). The lysate was sonicated for nine pulses, on ice, until no longer viscous. Post sonication, lysates were centrifuged for 5 min at 10,000 RCF at 4 °C. 800 μL of lysate containing AHA-labeled proteins were covalently coupled to alkyne-tagged agarose beads using reagents provided in the kit. Beads were washed repeatedly with SDS (1% SDS, 100 mM Tris pH 8, 250 mM NaCl and 5 mM EDTA) and alkyne-bound proteins were reduced with DTT at 70 °C before alkylation with 1 mL of 41 mM iodoacetamide for 30 mins, protected from light at room temperature. Beads were then washed sequentially to remove non-specifically bound proteins with 100 column volume SDS wash buffer, 8 M urea and finally with 20% acetonitrile. Bound proteins were digested on-resin with 1 μg trypsin (Trypsin Gold, Mass spectrometry grade, Promega) at 37 °C overnight in 25 mM ammonium bicarbonate, and the resulting tryptic peptides were desalted using two layers of hand-packed StageTips^80^. Desalted peptides were dried to a small droplet, under vacuum, in a SpeedVac and reconstituted in 12 μL of 4% formic acid supplemented with 2% acetonitrile. Five microlitres of the final peptide solution was used for the nano LC-MS/MS analysis.

### Liquid chromatography coupled to Tandem Mass Spectrometry (LC-MS/MS)

LC-MS/MS was conducted using a Thermo Scientific EASY-nLC 1000 coupled to a Q Exactive, High Field mass spectrometer (ThermoFisher Scientific) equipped with a nanoelectrospray ionization source. Peptide separation was achieved with a self-packed ReproSil-Pur C18 AQ 3μ reverse phase column (Dr.Maisch GmbH, Germany, 75 μm inner ID, ~25 cm long). Peptides were eluted via a gradient of 3–40% acetonitrile in 0.1% formic acid over 120 min at a flow rate of 250 nL/min at 45 °C, maintained with a column heater (Sonation GmbH, Germany). The mass spectrometer was operated in data-dependent mode with survey scans (MS) acquired at a resolution of 120,000 at *m/z* 400. Up to the top 15 most abundant precursors from the survey scan were selected with an isolation window of 1.6 Thomsons and fragmented by higher energy collisional dissociation with normalized collision energies of 27. The maximum ion injection times for the survey scan and the MS/MS scans were 60 ms, and the ion target value for both scan modes were set to 1,000,000.

### Protein identification and quantitation

Raw files obtained from mass spectrometry runs were processed using the MaxQuant computational proteomics platform (Version 1.5.5.1^81^) for peptide identification and quantitation. Fragmentation spectra were searched against the Uniprot mouse protein database (downloaded on 12/20/2017, containing 16,950 non-redundant protein entries, combined with 262 common contaminants), allowing for up to two missed tryptic cleavages. Cysteine carbamidomethylation was set as a fixed modification, and methionine oxidation, acetylation of protein N-terminal, Lysine-4, Lysin-8, Arginine-6 and Arginine-10 were used as variable modifications for the database search. The mass tolerances were set to 7 ppm for precursor, and 10 ppm for fragment, respectively. A false discovery rate (FDR) of 1% was applied to both peptide and protein identifications.

### Computational processing of BONLAC MS data

Quantification reproducibility between replicates as determined by correlation between peptide intensity measurements within each experiment are presented in Supplementary Fig. 11 and Supplementary Fig. 12. Normalized heavy vs. medium (H/M) ratios were obtained from MaxQuant. For experiments in which isotopic labelling was reversed, ratios were inverted.

### Protein identity and interaction interpretation

Gene Ontology analysis was performed using the Database for Annotation, Visualization and Integrated Discovery (DAVID version 6.8)^82^. Candidate proteins selected according to the methods outlined above were added to the software as a ‘gene list’, and the background was considered as all proteins previously measured in hippocampal brain slices^23^. To depict the function clustering of the data described by DAVID, the STRING database (version 11.0) was used. This online resource contains both known and predicted protein–protein interactions^83^. Clusters of proteins of interest were highlighted for visualization, and FDR of GO term was noted.

For hierarchical clustering of datasets, Cytoscape (Version 3.71) was used. Datasets were uploaded to the program as lists of proteins, and STRING enrichment was conducted against the mouse genome. Fold change (average or individual APP/PS1:WT samples, as appropriate) were uploaded against the relevant network. Hierarchical clusters were generated using the clusterMaker plug-in, with pairwise average linkage and Euclidean distance metric.

### Western blot validation

All western blotting was carried out as previously described^84^. Briefly, hippocampal tissue was homogenized in lysis buffer protease and phosphatase inhibitors. Protein concentration was measured using BCA assay (Pierce). Aliquots of protein (20–40 μg) were separated using Bolt Bis-Tris gels (4–12%; Thermo Scientific) and transferred to a nitrocellulose membrane. Membranes were washed and probed for total protein using MemCode Reversible Protein Stain (Thermo Scientific), before the expression of the protein of interest was probed using appropriate primary and secondary antibody pairs. All primary antibodies were incubated with the membranes overnight at 4 °C, rocking gently. All secondary antibodies were incubated with the membranes for 1 h at room temperature. Antibody signal was detected using chemiluminescence (GE Healthcare). Density of bands corresponding to the specified molecular weight for each target protein were normalized to the density of individual lanes of total protein as detected using MemCode or Ponceau staining.

### Antibodies

For immunohistochemistry rabbit anti-amyloid beta antibody was used (1:200; clone 6E10, ENZ-ABS612-0200, lot # 01172006, Enzo Life Sciences). The following antibodies were used in the western blot analyses presented in this study: rabbit anti-APP monoclonal antibody (1:1000; clone Y188, ab32136, lot # GR3248334-7, Abcam), rabbit anti-EAAT1 polyclonal antibody (1:1000; Ab416, lot # GR3328985-3, Abcam), rabbit anti-GAP-43 polyclonal antibody (1:500; ab5220, lot # 3245135, Sigma), rabbit anti-Hsp1a1 polyclonal antibody (1:1000; AV33096, lot # QC2724, Sigma–Aldrich), rabbit anti-Rpl13 polyclonal antibody (1:1000; PA5-41715, lot # UD2757834A, Thermofisher) and rabbit anti-Rpl18 polyclonal antibody (1:5000; ab241988, lot # GR3304969-2, Abcam). Secondary antibodies were either goat anti-mouse IgG HRP or goat anti-rabbit IgG HRP (Promega; 1:10,000), respectively.

### Statistics and reproducibility

Investigators were blinded to animal genotype during data collection and analysis.

#### BONLAC analysis

Our group has extensive experience in performing BONLAC analysis, which was developed in house. We have employed this method in a variety of systems, including in vitro and ex vivo approaches. Thus, we based our calculations on previous analyses published in this lab, in which a minimum of three mass spectrometry runs/condition were used^23,28^. In this current analysis, we performed 5–7 mass spectrometry runs/condition. Seven replicates per age group were analyzed using BONLAC and mass spectrometry. Each sample was composed of equal numbers of hippocampal slices from one biologically independent APP/PS1 mouse and one age-matched biologically independent WT mouse, labelled with AHA and SILAC labels simultaneously. Following labelling, slices were pooled, snap frozen and processed for mass spectrometry analysis. Two samples were excluded from the mass spectrometry analysis of young (3–5 month old) mice. The first sample was composed of hippocampal slices from one WT and one APP/PS1 mouse, where the APP/PS1 slices had visibly degraded during the labeling process. The second sample was excluded as the medium-heavy SILAC labeling ratio generated via mass spectrometry as the average incorporation relative to all other samples was 10-fold higher, indicating an abnormality occurred during sample processing; thus, five replicates were examined for the 3–5-month-old group, and seven replicates were examined for the 12+-month-old group. Reproducibility, as shown via correlation scatterplots and Pearson correlation value calculations are available for the young and aged datasets in Supplementary Fig. 11.

Following MaxQuant automated data processing, the list of proteins and ratios was run through a custom-made *R* script to identify early protein candidates where the average ratio was above or below a threshold of 20% (≥0.8 or ≤1.2). This 20% threshold for identification was selected as reported changes in this range can be reliably detected by western blot and this threshold has been used previously^85–88^. Putative candidate proteins were isolated from this non-biased screen if a protein ratio was detected in the majority of samples (≥3/5 young samples and ≥5/7 aged samples). This requirement was based on previously reported data analysis strategies^85–87^. Candidate proteins were finalized if the majority of samples showed a shift in de novo synthesis in the same direction (majority increased or majority decreased). This approach has been previously published by Bowling et al.^86^. This method allows for non-biased selection of up- or downregulated proteins, taking variation between replicates into account. Although not considered in this analysis, Significance B analyses are provided as Supplementary Data 2.

#### Western blot analysis

Hippocampal lysate from a minimum of five animals per genotype and per age group were run for western blot analysis. Mean band densities for each protein of interest were quantified using ImageStudioLite (Version 5.2.5) and normalized to total protein expression within the entire lane as measured by MemCode or Ponceau staining. Representative uncut western blot membrane images for each protein of interest and corresponding total protein stain images are available in Supplemental Fig. 13. Normalized data from APP/PS1 samples was expressed relative to corresponding samples from WT animals within each blot to allow comparison across blots. Significance between expression in WT versus APP/PS1 hippocampal tissue was determine using GraphPad Prism (version 8) with unpaired two-tailed *t*-tests.

### Reporting summary

Further information on research design is available in the Nature Research Reporting Summary linked to this article.

## Supplementary information

Supplementary Information

Description of Additional Supplementary Files

Supplementary Data 1

Supplementary Data 2

Reporting Summary

## Data Availability

The raw mass spectrometry data generated during this study are available at MassIVE (https://massive.ucsd.edu/ProteoSAFe/static/massive.jsp; Center for Computational Mass Spectrometry, University of California, San Diego) with the accession number (MSV000085962)^89^. Uncropped western blot images for Fig. 4 are presented in Supplementary Fig. 13. Data underlying the main figures are presented in Supplementary Data 1. All other data supporting the findings of this study are available either within the body of the manuscript, within the supplementary information files, or are available from the corresponding author upon reasonable request.

## Code availability

The custom-made *R* script for unbiased identification of potential candidate proteins based on MaxQuant normalized H/M ratios is available from the corresponding author.

## References

[1] Alzheimer A (1907). Uber eine eigenartige Erkrankung der Hirnrinde. Zentralbl. Nervenh. Psych..

[2] Scahill RI, Schott JM, Stevens JM, Rossor MN, Fox NC (2002). Mapping the evolution of regional atrophy in Alzheimer’s disease: unbiased analysis of fluid-registered serial MRI. Proc. Natl Acad. Sci. USA.

[3] Hernández‐Ortega K, Garcia‐Esparcia P, Gil L, Lucas JJ, Ferrer I (2016). Altered machinery of protein synthesis in Alzheimer’s: from the nucleolus to the ribosome. Brain Pathol..

[4] Kaushik S, Cuervo AM (2015). Proteostasis and aging. Nat. Med..

[5] Ciechanover A, Kwon YT (2015). Degradation of misfolded proteins in neurodegenerative diseases: therapeutic targets and strategies. Exp. Mol. Med..

[6] Keller JN, Hanni KB, Markesbery WR (2000). Impaired proteasome function in Alzheimer’s disease. J. Neurochem..

[7] Mawuenyega KG (2010). Decreased clearance of CNS β-amyloid in Alzheimer’s disease. Science.

[8] Holt CE, Martin KC, Schuman EM (2019). Local translation in neurons: visualization and function. Nat. Struct. Mol. Biol..

[9] Richter JD, Klann E (2009). Making synaptic plasticity and memory last: mechanisms of translational regulation. Genes Dev..

[10] Costa-Mattioli M, Sossin WS, Klann E, Sonenberg N (2009). Translational control of long-lasting synaptic plasticity and memory. Neuron.

[11] An W-L (2003). Up-regulation of phosphorylated/activated p70 S6 kinase and its relationship to neurofibrillary pathology in Alzheimer’s disease. Am. J. Pathol..

[12] Chang RC, Wong AK, Ng H-K, Hugon J (2002). Phosphorylation of eukaryotic initiation factor-2α (eIF2α) is associated with neuronal degeneration in Alzheimer’s disease. Neuroreport.

[13] Ferrer I (2002). Differential expression of phosphorylated translation initiation factor 2 alpha in Alzheimer’s disease and Creutzfeldt–Jakob’s disease. Neuropathol. Appl. Neurobiol..

[14] Li X (2004). Phosphorylated eukaryotic translation factor 4E is elevated in Alzheimer brain. Neuroreport.

[15] Ma T (2013). Suppression of eIF2α kinases alleviates Alzheimer’s disease–related plasticity and memory deficits. Nat. Neurosci..

[16] Lourenco MV (2013). TNF-α mediates PKR-dependent memory impairment and brain IRS-1 inhibition induced by Alzheimer’s β-amyloid oligomers in mice and monkeys. Cell Metab..

[17] Ding Q, Markesbery WR, Chen Q, Li F, Keller JN (2005). Ribosome dysfunction is an early event in Alzheimer’s disease. J. Neurosci..

[18] Langstrom N, Anderson J, Lindroos H, Winbland B, Wallace W (1989). Alzheimer’s disease-associated reduction of polysomal mRNA translation. Mol. Brain Res..

[19] Sajdel-Sulkowska EM, Marotta CA (1984). Alzheimer’s disease brain: alterations in RNA levels and in a ribonuclease-inhibitor complex. Science.

[20] Buffington SA, Huang W, Costa-Mattioli M (2014). Translational control in synaptic plasticity and cognitive dysfunction. Annu. Rev. Neurosci..

[21] Evans HT, Benetatos J, van Roijen M, Bodea LG, Götz J (2019). Decreased synthesis of ribosomal proteins in tauopathy revealed by non‐canonical amino acid labelling. EMBO J..

[22] Ma Y, McClatchy DB, Martínez-Bartolomé S, Bamberger C, Yates JR (2021). Temporal quantitative profiling of newly synthesized proteins during Aβ accumulation. J. Proteome Res..

[23] Bowling H (2016). BONLAC: a combinatorial proteomic technique to measure stimulus-induced translational profiles in brain slices. Neuropharmacology.

[24] Zhang G (2014). In-depth quantitative proteomic analysis of de novo protein synthesis induced by brain-derived neurotrophic factor. J. Proteome Res..

[25] Eichelbaum K, Winter M, Berriel Diaz M, Herzig S, Krijgsveld J (2012). Selective enrichment of newly synthesized proteins for quantitative secretome analysis. Nat. Biotechnol..

[26] Dieterich DC, Link AJ, Graumann J, Tirrell DA, Schuman EM (2006). Selective identification of newly synthesized proteins in mammalian cells using bioorthogonal noncanonical amino acid tagging (BONCAT). Proc. Natl Acad. Sci. USA.

[27] Ong SE (2002). Stable isotope labeling by amino acids in cell culture, SILAC, as a simple and accurate approach to expression proteomics. Mol. Cell Proteomics.

[28] Bowling H (2019). Altered steady state and activity-dependent de novo protein expression in fragile X syndrome. Nat. Commun..

[29] Volianskis A, Køstner R, Mølgaard M, Hass S, Jensen MS (2010). Episodic memory deficits are not related to altered glutamatergic synaptic transmission and plasticity in the CA1 hippocampus of the APPswe/PS1δE9-deleted transgenic mice model of ß-amyloidosis. Neurobiol. Aging.

[30] Beckelman BC (2019). Genetic reduction of eEF2 kinase alleviates pathophysiology in Alzheimer’s disease model mice. J. Clin. Invest.

[31] Ma, Y., McClatchy, D. B., Martínez-Bartolomé, S., Bamberger, C. & Yates, J. R. A temporal quantitative profiling of newly synthesized proteins during Aβ accumulation. *bioRxiv*10.1101/2020.01.14.906560 (2020). 10.1021/acs.jproteome.0c00645 PMC7775911 33147027

[32] Radde R (2006). Abeta42-driven cerebral amyloidosis in transgenic mice reveals early and robust pathology. EMBO Rep..

[33] Rallis A (2020). Hedgehog signaling modulates glial proteostasis and lifespan. Cell Rep..

[34] Bogdanovic N, Davidsson P, Volkmann I, Winblad B, Blennow K (2000). Growth-associated protein GAP-43 in the frontal cortex and in the hippocampus in Alzheimer’s disease: an immunohistochemical and quantitative study. J. Neural Transm..

[35] de la Monte SM, Ng SC, Hsu DW (1995). Aberrant GAP-43 gene expression in Alzheimer’s disease. Am. J. Pathol..

[36] Alonso-Nanclares L, Merino-Serrais P, Gonzalez S, DeFelipe J (2013). Synaptic changes in the dentate gyrus of APP/PS1 transgenic mice revealed by electron microscopy. J. Neuropathol. Exp. Neurol..

[37] Minkeviciene R (2009). Amyloid beta-induced neuronal hyperexcitability triggers progressive epilepsy. J. Neurosci..

[38] Gengler S, Hamilton A, Hölscher C (2010). Synaptic plasticity in the hippocampus of a APP/PS1 mouse model of Alzheimer’s disease is impaired in old but not young mice. PLoS ONE.

[39] Serneels L (2009). gamma-Secretase heterogeneity in the Aph1 subunit: relevance for Alzheimer’s disease. Science.

[40] Park JH (2006). Subcutaneous nogo receptor removes brain amyloid-β and improves spatial memory in Alzheimer’s transgenic mice. J. Neurosci..

[41] Xu Z-Q (2013). Aerobic exercise combined with antioxidative treatment does not counteract moderate- or mid-stage Alzheimer-like pathophysiology of APP/PS1 mice. CNS Neurosci. Ther..

[42] Gruart A, López-Ramos JC, Muñoz MD, Delgado-García JM (2008). Aged wild-type and APP, PS1, and APP + PS1 mice present similar deficits in associative learning and synaptic plasticity independent of amyloid load. Neurobiol. Dis..

[43] Trinchese F (2004). Progressive age‐related development of Alzheimer‐like pathology in APP/PS1 mice. Ann. Neurol..

[44] Pras, A. & Nollen, E. A. A. Regulation of age-related protein toxicity. *Front. Cell Dev. Biol.*10.3389/fcell.2021.637084 (2021). 10.3389/fcell.2021.637084 PMC7973223 33748125

[45] Smith DL, Pozueta J, Gong B, Arancio O, Shelanski M (2009). Reversal of long-term dendritic spine alterations in Alzheimer disease models. Proc. Natl Acad. Sci. USA.

[46] Zhang H (2005). Synaptic fatigue is more pronounced in the APP/PS1 transgenic mouse model of Alzheimer’s disease. Curr. Alzheimer Res..

[47] Hirai K (2001). Mitochondrial abnormalities in Alzheimer’s disease. J. Neurosci..

[48] Baloyannis SJ (2006). Mitochondrial alterations in Alzheimer’s disease. J. Alzheimer’s Dis..

[49] Swerdlow RH (2018). Mitochondria and mitochondrial cascades in Alzheimer’s disease. J. Alzheimers Dis..

[50] Cataldo AM (1995). Gene expression and cellular content of cathepsin D in Alzheimer’s disease brain: evidence for early up-regulation of the endosomal-lysosomal system. Neuron.

[51] Nixon RA (2017). Amyloid precursor protein and endosomal-lysosomal dysfunction in Alzheimer’s disease: inseparable partners in a multifactorial disease. FASEB J..

[52] Tomljanovic Z, Patel M, Shin W, Califano A, Teich AF (2018). ZCCHC17 is a master regulator of synaptic gene expression in Alzheimer’s disease. Bioinformatics.

[53] Chang W-L (2003). Molecular characterization of a novel nucleolar protein, pNO40. Biochem. Biophys. Res. Commun..

[54] Shi Z (2017). Heterogeneous ribosomes preferentially translate distinct subpools of mRNAs genome-wide. Mol. Cell.

[55] Kong W (2009). Independent component analysis of Alzheimer’s DNA microarray gene expression data. Mol. Neurodegener..

[56] Martínez-Ballesteros M, García-Heredia JM, Nepomuceno-Chamorro IA, Riquelme-Santos JC (2017). Machine learning techniques to discover genes with potential prognosis role in Alzheimer’s disease using different biological sources. Inf. Fusion.

[57] Singh AK, Pati U (2015). CHIP stabilizes amyloid precursor protein via proteasomal degradation and p53-mediated trans-repression of β-secretase. Aging Cell.

[58] Habib LK, Lee MTC, Yang J (2010). Inhibitors of catalase-amyloid interactions protect cells from beta-amyloid-induced oxidative stress and toxicity. J. Biol. Chem..

[59] Poirier Y, Grimm A, Schmitt K, Eckert A (2019). Link between the unfolded protein response and dysregulation of mitochondrial bioenergetics in Alzheimer’s disease. Cell. Mol. Life Sci..

[60] Chen L (2019). Studies on APP metabolism related to age-associated mitochondrial dysfunction in APP/PS1 transgenic mice. Aging (Albany NY).

[61] Hauptmann S (2009). Mitochondrial dysfunction: an early event in Alzheimer pathology accumulates with age in AD transgenic mice. Neurobiol. Aging.

[62] Moreira PI, Santos MS, Oliveira CR (2007). Alzheimer’s disease: a lesson from mitochondrial dysfunction. Antioxid. Redox Signal..

[63] Nunomura A (2001). Oxidative damage is the earliest event in Alzheimer disease. J. Neuropathol. Exp. Neurol..

[64] Du H, Guo L, Yan SS (2012). Synaptic mitochondrial pathology in Alzheimer’s disease. Antioxid. Redox Signal..

[65] Bo, H. et al. Exercise-induced neuroprotection of hippocampus in APP/PS1 transgenic mice via upregulation of mitochondrial 8-oxoguanine DNA glycosylase. *Oxid. Med. Cell. Longev.* **2014**, 834502 (2014). 10.1155/2014/834502 PMC4236906 25538817

[66] David DC (2005). Proteomic and functional analyses reveal a mitochondrial dysfunction in P301L tau transgenic mice. J. Biol. Chem..

[67] Minkeviciene R (2008). Age-related decrease in stimulated glutamate release and vesicular glutamate transporters in APP/PS1 transgenic and wild-type mice. J. Neurochem..

[68] Martins RN (2018). Alzheimer’s disease: a journey from amyloid peptides and oxidative stress, to biomarker technologies and disease prevention strategies—gains from AIBL and DIAN cohort studies. J. Alzheimer’s Dis..

[69] Markesbery WR (2010). Neuropathologic alterations in mild cognitive impairment: a review. J. Alzheimer’s Dis..

[70] Landau, S. M. & Frosch, M. P. Tracking the earliest pathologic changes in Alzheimer disease. (AAN Enterprises, 2014). 10.1212/WNL.0000000000000392 24706009

[71] Yoshihama M (2002). The human ribosomal protein genes: sequencing and comparative analysis of 73 genes. Genome Res..

[72] Slavov N, Semrau S, Airoldi E, Budnik B, van Oudenaarden A (2015). Differential stoichiometry among core ribosomal proteins. Cell Rep..

[73] Shigeoka T (2019). On-site ribosome remodeling by locally synthesized ribosomal proteins in axons. Cell Rep..

[74] Ximerakis M (2019). Single-cell transcriptomic profiling of the aging mouse brain. Nat. Neurosci..

[75] McIntosh KB, Bhattacharya A, Willis IM, Warner JR (2011). Eukaryotic cells producing ribosomes deficient in Rpl1 are hypersensitive to defects in the ubiquitin-proteasome system. PLoS ONE.

[76] Zecha J (2019). TMT labeling for the masses: a robust and cost-efficient, in-solution labeling approach. Mol. Cell. Proteomics.

[77] Chévere-Torres I (2012). Metabotropic glutamate receptor-dependent long-term depression is impaired due to elevated ERK signaling in the ΔRG mouse model of tuberous sclerosis complex. Neurobiol. Dis..

[78] Kaphzan H (2013). Genetic reduction of the α1 subunit of Na/K-ATPase corrects multiple hippocampal phenotypes in Angelman syndrome. Cell Rep..

[79] Santini E (2015). Mitochondrial superoxide contributes to hippocampal synaptic dysfunction and memory deficits in Angelman syndrome model mice. J. Neurosci..

[80] Rappsilber J, Mann M, Ishihama Y (2007). Protocol for micro-purification, enrichment, pre-fractionation and storage of peptides for proteomics using StageTips. Nat. Protoc..

[81] Cox J, Mann M (2008). MaxQuant enables high peptide identification rates, individualized ppb-range mass accuracies and proteome-wide protein quantification. Nat. Biotechnol..

[82] Sherman BT, Lempicki RA (2009). Systematic and integrative analysis of large gene lists using DAVID bioinformatics resources. Nat. Protoc..

[83] Szklarczyk D (2019). STRING v11: protein-protein association networks with increased coverage, supporting functional discovery in genome-wide experimental datasets.. Nucleic Acids Res.

[84] Sharma A (2010). Dysregulation of mTOR signaling in fragile X syndrome. J. Neurosci..

[85] Hodas JJ (2012). Dopaminergic modulation of the hippocampal neuropil proteome identified by bioorthogonal noncanonical amino acid tagging (BONCAT). Proteomics.

[86] Bowling H (2014). Antipsychotics activate mTORC1-dependent translation to enhance neuronal morphological complexity. Sci. Signal.

[87] Spellman DS, Deinhardt K, Darie CC, Chao MV, Neubert TA (2008). Stable isotopic labeling by amino acids in cultured primary neurons: application to brain-derived neurotrophic factor-dependent phosphotyrosine-associated signaling. Mol. Cell. Proteomics.

[88] Butko MT (2013). In vivo quantitative proteomics of somatosensory cortical synapses shows which protein levels are modulated by sensory deprivation. Proc. Natl Acad. Sci. USA.

[89] Elder, M. K. et al. Dysregulation of the de novo proteome accompanies pathology progression in the APP/PS1 mouse model. MassIVE https://massive.ucsd.edu/ProteoSAFe/dataset.jsp?task=b15077d9187447cb8388dae7b9766fb2 (2021).

