# Supplementary material for: Age-dependent shift in the de novo proteome accompanies pathogenesis in an Alzheimer’s disease mouse model
Source: Commun Biol. 2021 Jun 30;4:823. doi: 10.1038/s42003-021-02324-6 (PMC8245541; doi:10.1038/s42003-021-02324-6)
Supplement: Supplementary file 1 — Supplementary Information [file 42003_2021_2324_MOESM1_ESM.pdf]

# **Age-dependent shift in the *de novo* proteome accompanies pathogenesis in an Alzheimer's disease mouse model**

Elder et al.,

Supplementary Figures and Tables

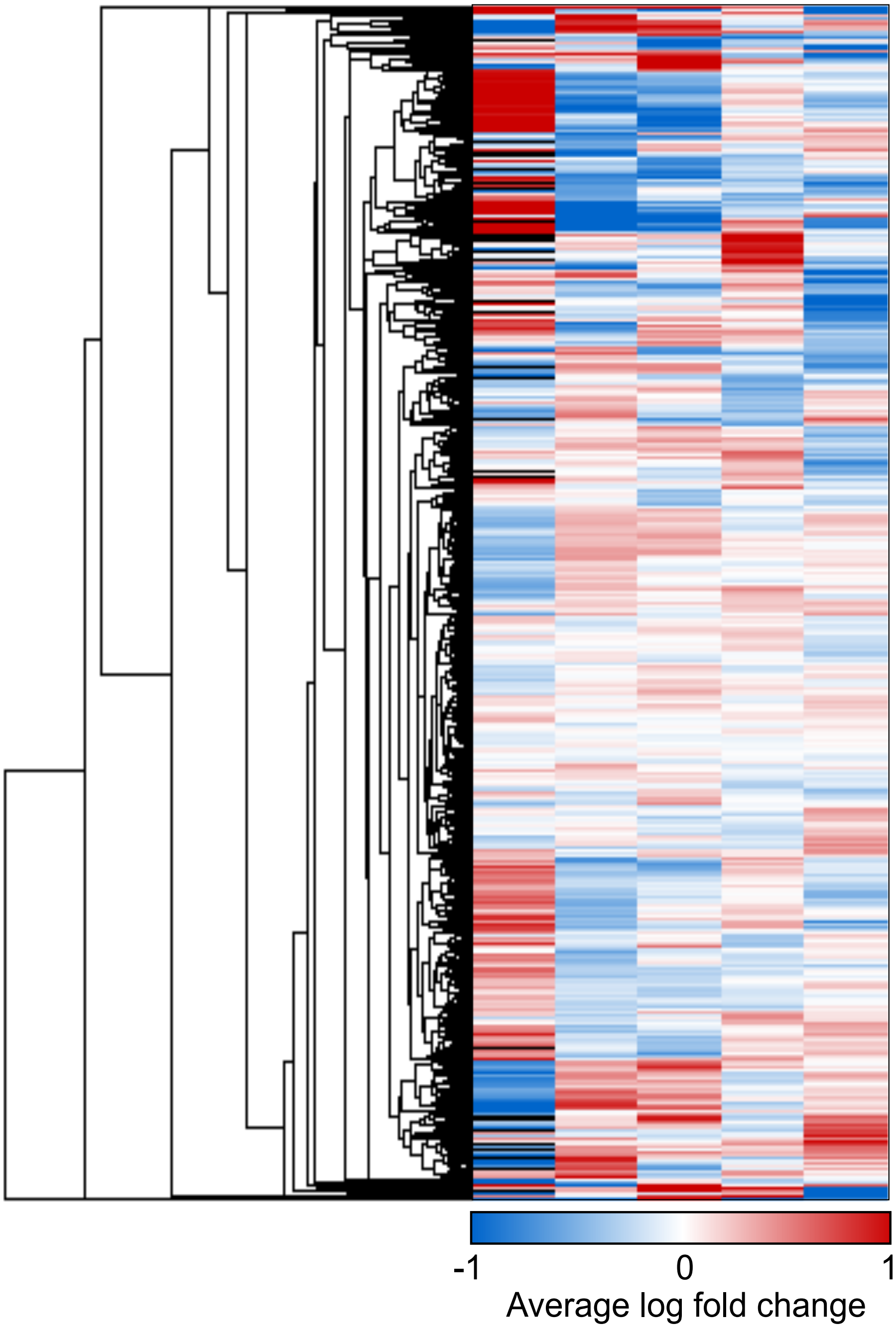


**Supplementary Figure 1. All proteins detected in the young APP/PS1 hippocampus as detected by BONLAC.** Hierarchical clustering-generated heatmap showing all protein log fold-change ratios identified in at least one sample from the BONLAC screen. Red indicates proteins who show higher levels of *de novo* synthesis in 3-5 month-old APP/PS1 mice compared to wild-type (WT) littermates, while downregulated proteins are shown in blue. White = no change. Black = MaxQuant ratio not calculated. *n* = 5 biologically independent samples.

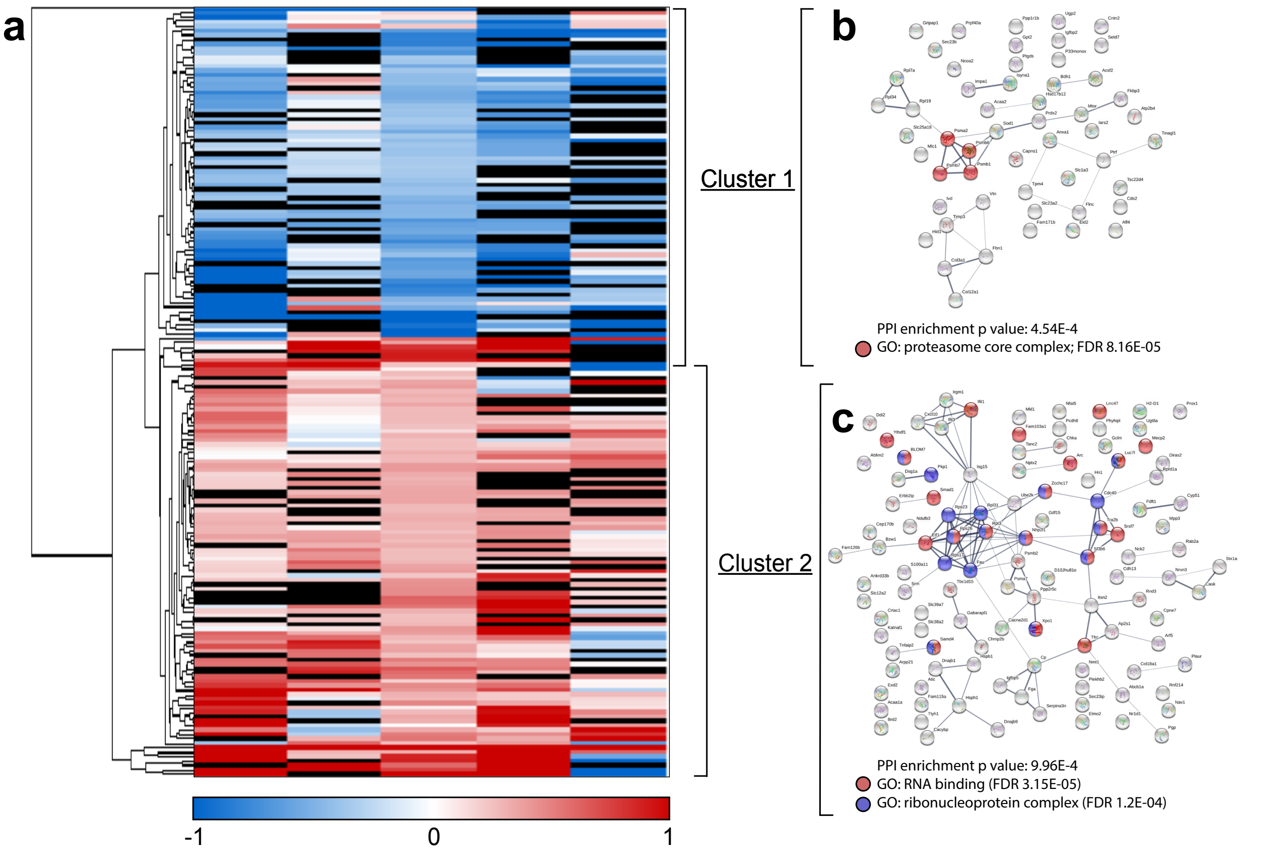


**Supplementary Figure 2. Dysregulated protein candidates in the young APP/PS1 hippocampus as detected by BONLAC.**
**a)** Hierarchical clustered heatmap showing log fold change of all candidate proteins identified from the BONLAC screen of 3-5 months-old APP/PS1 vs. wild-type (WT) ranked by automated C-score. Protein candidates were identified by *R* script as showing an average fold change across all samples of ± 20% (<0.8 or >1.2). Manual validation confirmed candidates were detected in the majority of samples (>3 out of 5 biologically independent samples), and that majority of samples showed the same trend (>50% of samples either <0.79 or >1.19). Nodes in blue indicate downregulation, while red indicates increased *de novo* synthesis in the young APP/PS1 mice compared to WT littermates. **b)** String diagram of Cluster 1 with key pathways highlighted. **c)** String diagram of Cluster 2 with key pathways highlighted. White = no change. Black = MaxQuant ratio not calculated. *n* = 5 biologically independent samples.

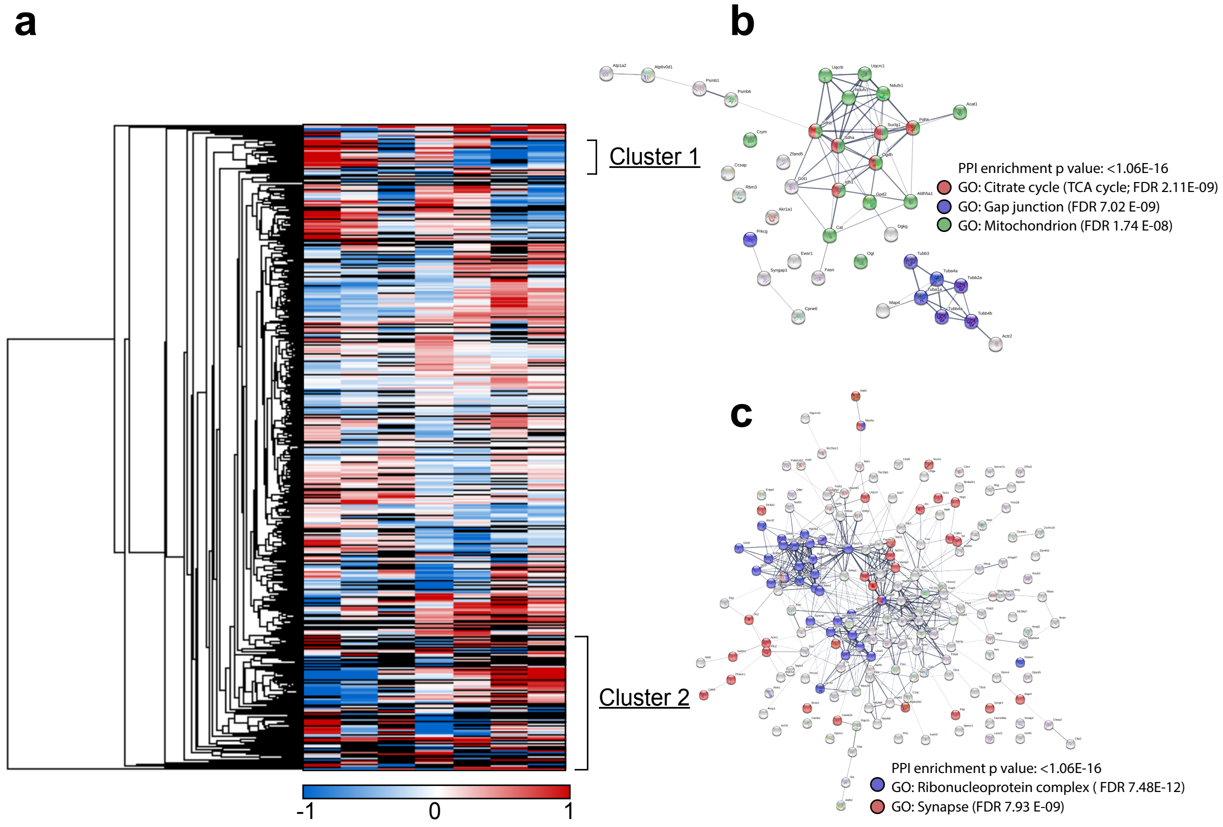


**Supplementary Figure 3. All proteins detected in the aged APP/PS1 hippocampus as detected by BONLAC. a)** Hierarchical clustering heatmap showing all protein log fold-change ratios identified in the majority of samples (>4 out of 7 biologically independent samples) from the BONLAC screen. Red indicates proteins which show higher levels of *de novo* synthesis in 12+ month-old APP/PS1 mice compared to WT littermates (APP is highlighted in top left), while downregulated proteins are shown in blue. **b)** String diagram of Cluster 1 with key pathways highlighted. **c)** String diagram of Cluster 2 with key pathways highlighted. White = no change. Black = MaxQuant ratio not calculated. *n* = 7 biologically independent samples.

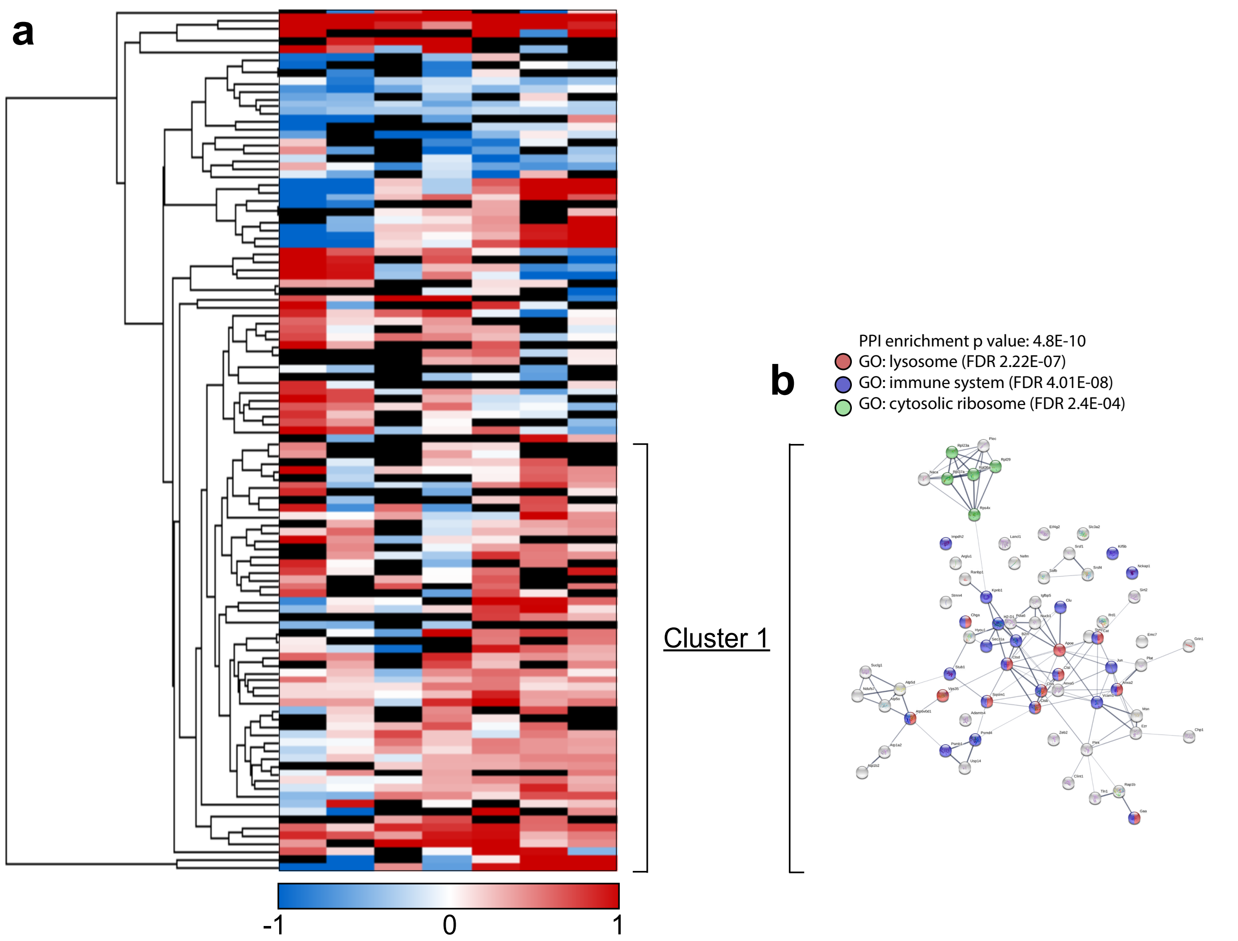


**Supplementary Figure 4. Dysregulated protein candidates in the hippocampus of aged APP/PS1 mice as detected by BONLAC. a)** Hierarchical clustered heatmap of candidate protein log fold changes identified from the BONLAC screen ranked by automated C-score. Protein candidates were identified by customized *R* script as showing an average fold change across all samples of ± 20% (<0.8 or >1.2). Manual validation confirmed candidates were detected in the majority of samples (>4 out of 7 biologically independent samples), and that majority of samples showed the same trend (>50% either <0.79 or >1.19). **b)** String diagram of Cluster 1with key pathways highlighted. Black cells indicate absence of ratio. Red = upregulated proteins. Blue = downregulated proteins. White = no change. Black = MaxQuant ratio not calculated.

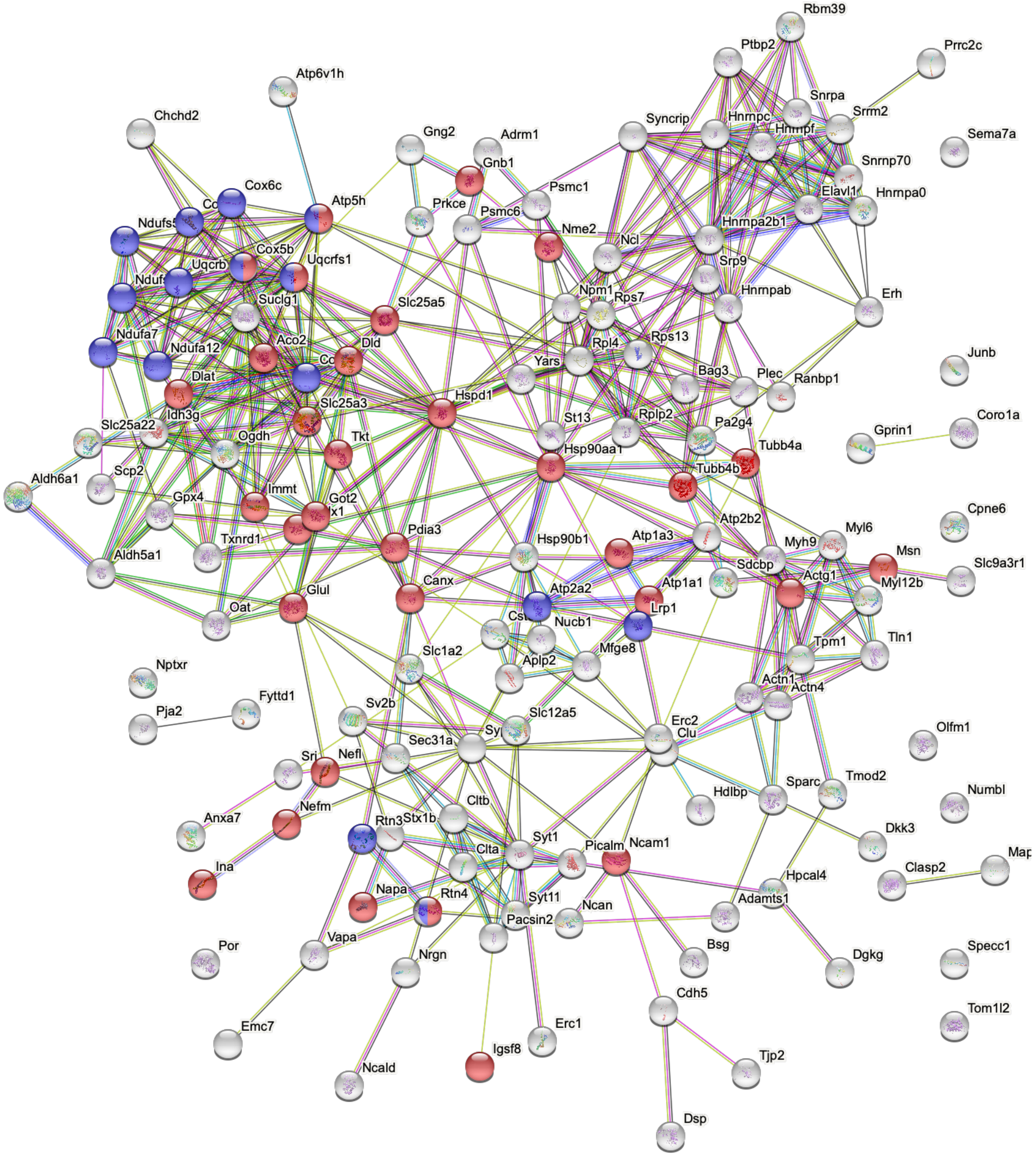


**Supplementary Figure 5. Visual depiction of proteins in Hierarchical Cluster 5 (from Figure 5).** String diagram showing biological networks between the proteins identified in Cluster 5. Figure generated by StringDb; Red node = GO: myelin sheath (FDR: 5.17E-18); blue node = GO: Alzheimer's disease (FDR: 8.16E-07).

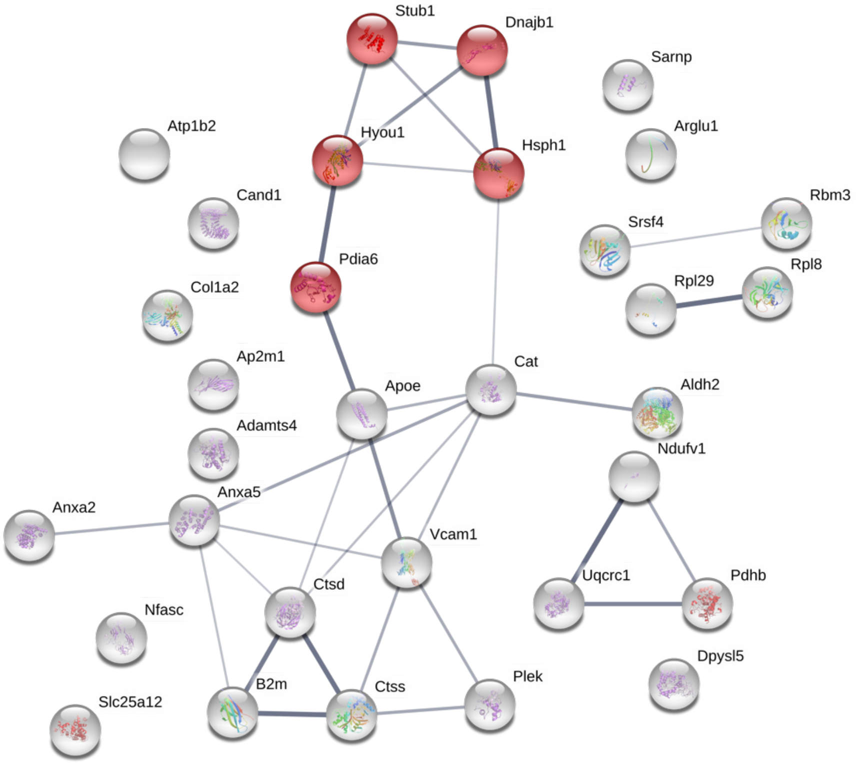


**Supplementary Figure 6. Visual depiction of proteins in Hierarchical Cluster 1 (from Figure 5).** String diagram showing biological networks between the proteins identified in Cluster 1. Figure generated by StringDb; Red node = GO: Protein processing in the endoplasmic reticulum; FDR:3.2E-4.

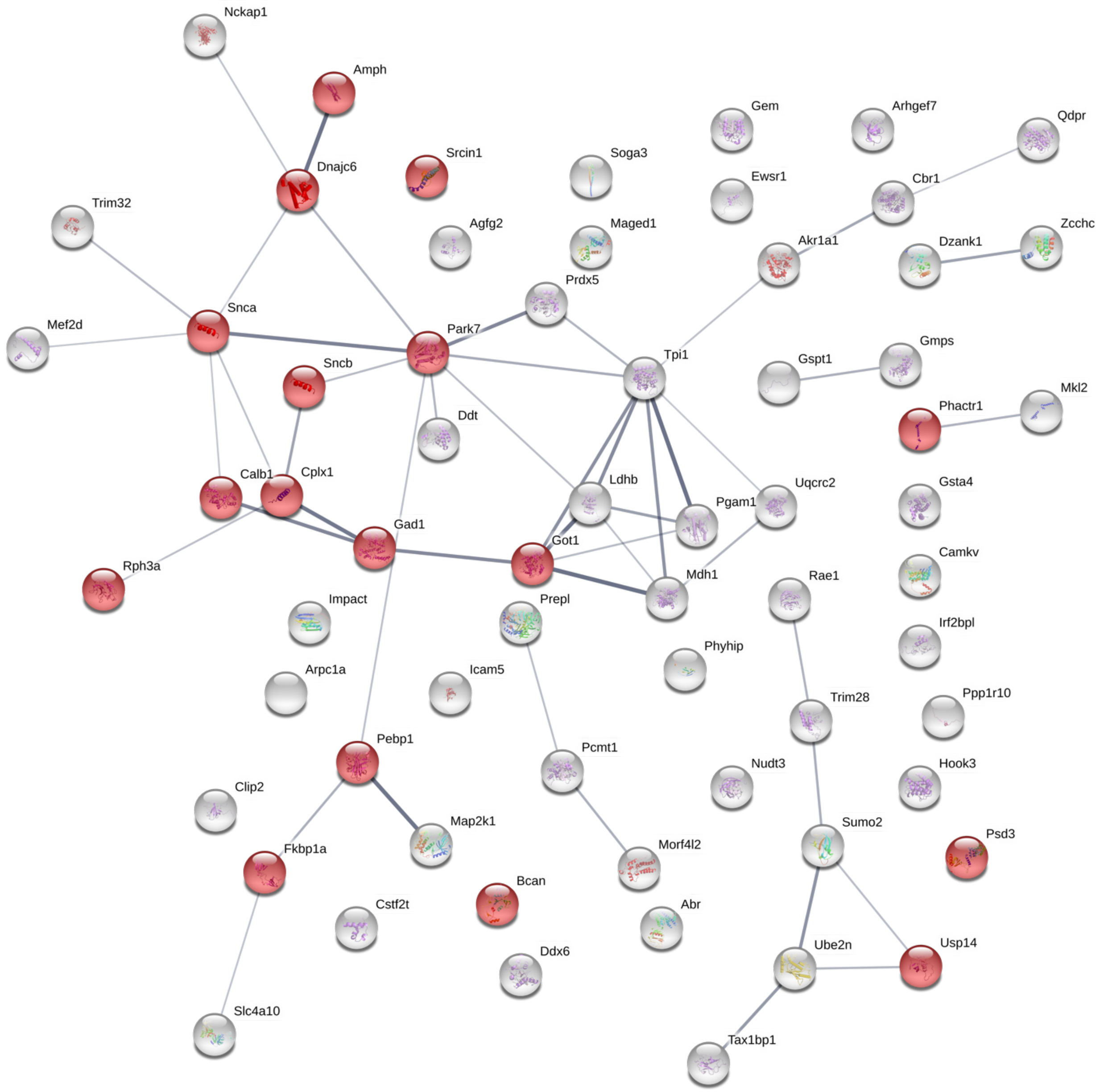


**Supplementary Figure 7. Visual depiction of proteins in Hierarchical Cluster 2 (from Figure 5).** String diagram showing biological networks between the proteins identified in Cluster 2. Figure generated by StringDb; Red node = GO: Synapse; FDR:5.6E-7.

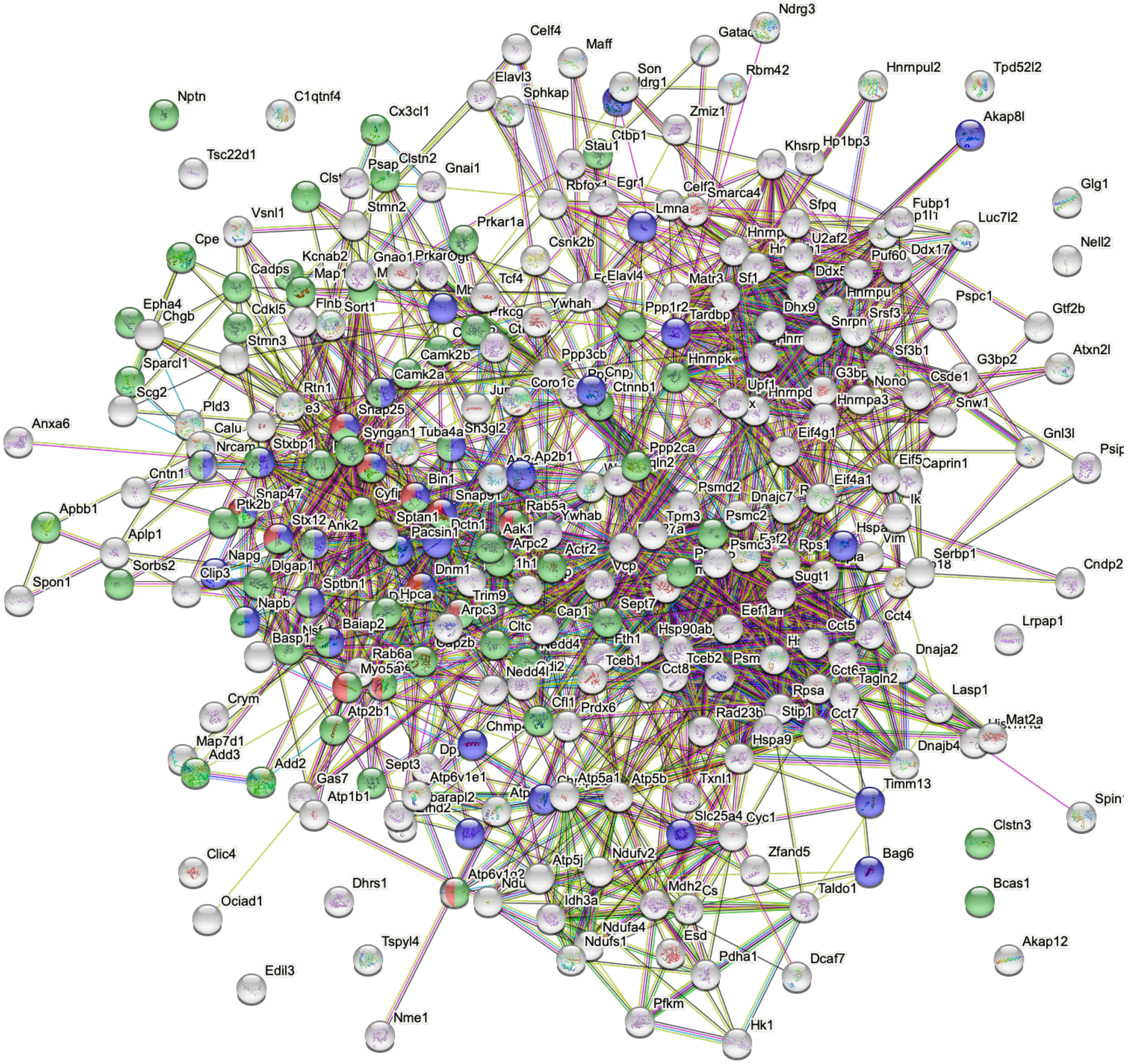


**Supplementary Figure 8. Visual depiction of proteins in Hierarchical Cluster 3 (from Figure 5).** String diagram showing biological networks between the proteins identified in Cluster 3. Figure generated by StringDb; Red node = GO: synaptic vesicle cycle (FDR: 2.77E-6); Green node = GO: synapse (FDR: 4.82E-29); Blue node = GO: membrane trafficking (FDR: 6.24E-11).

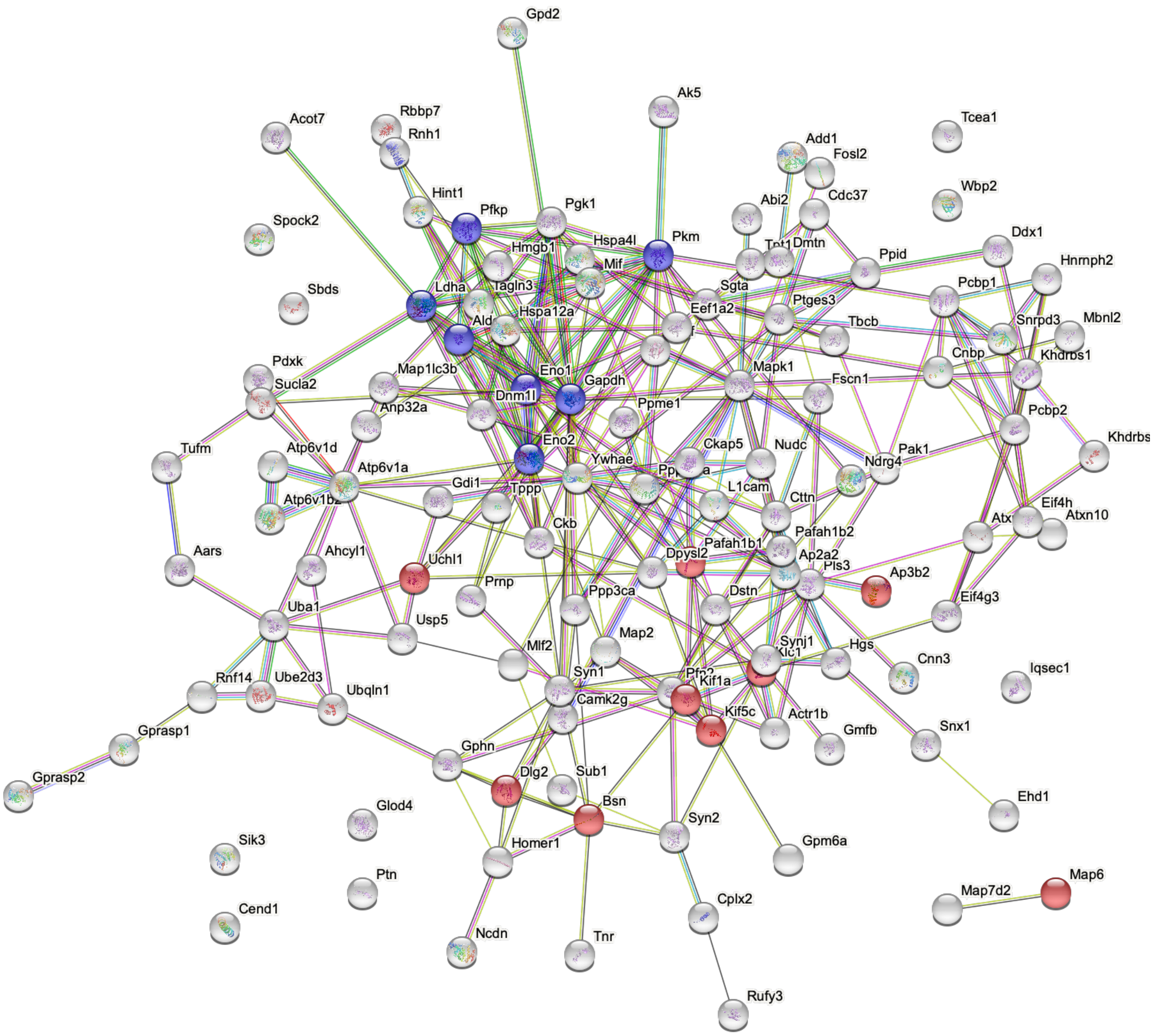


**Supplementary Figure 9. Visual depiction of proteins in Hierarchical Cluster 4 (from Figure 5).** String diagram showing biological networks between the proteins identified in Cluster 4. Figure generated by StringDb; Red node = GO: Axo-dendritic transport (FDR: 4.86E-8); Blue = GO: Glycolysis/Gluconeogenesis (FDR: 2.56E-5).

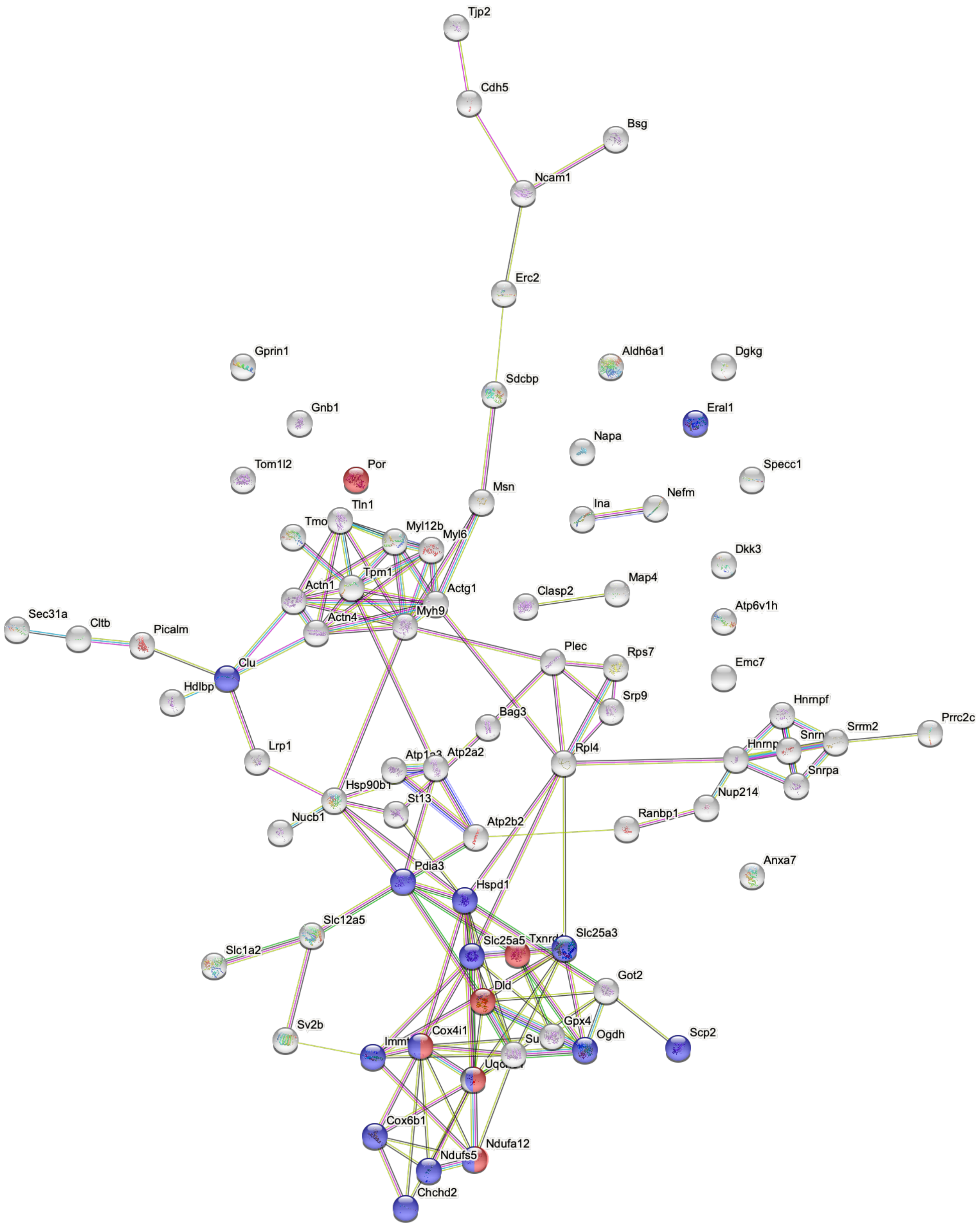


**Supplementary Figure 10. Visual depiction of proteins in Hierarchical Cluster 6 (from Figure 5).** String diagram showing biological networks between the proteins identified in Cluster 6. Figure generated by StringDb; Blue node = GO: mitochondrial part (FDR: 4.22E-07); Red node = GO: electron transfer activity (FDR: 2.88E-05).

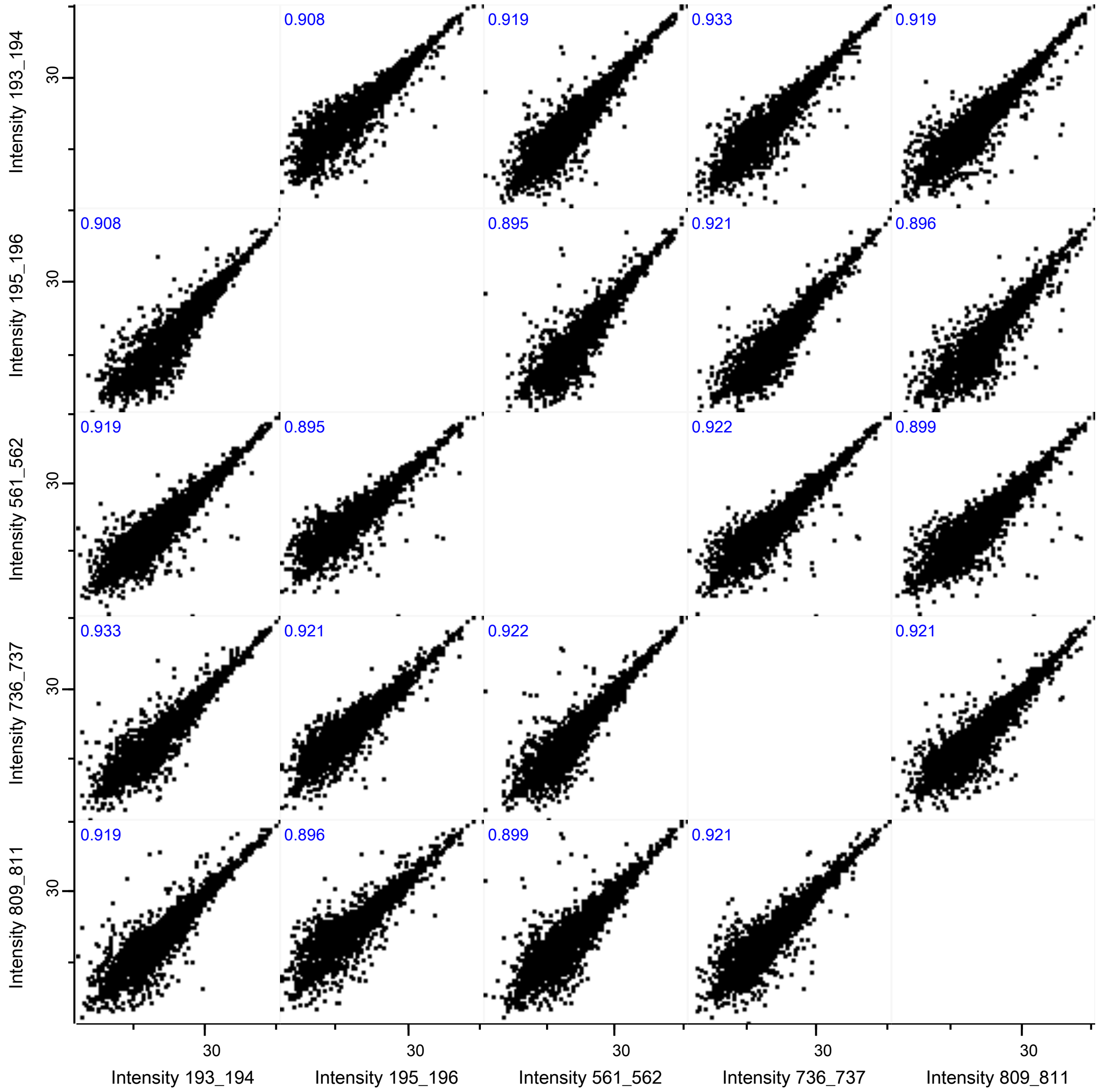


**Supplementary Figure 11. Quantification reproducibility between replicates within the young hippocampal de novo proteome study.** Correlation scatterplots and Pearson correlation value calculations for each SILAC pair (3-5 month old APP/PS1 and WT mice).

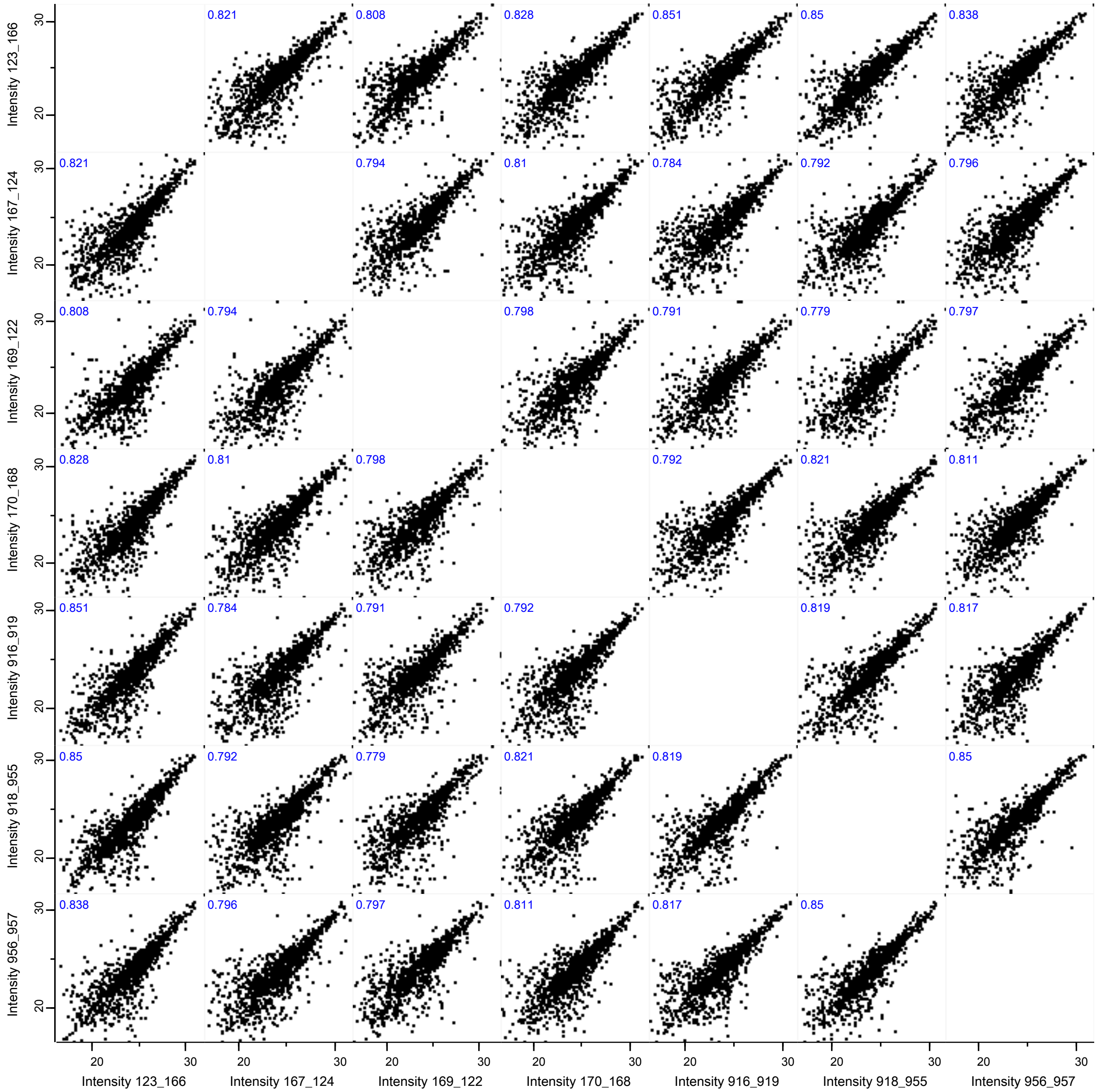


**Supplementary Figure 12. Quantification reproducibility between replicates within the aged hippocampal de novo proteome study.** Correlation scatterplots and Pearson correlation value calculations for each SILAC pair (>12 month old APP/PS1 and WT mice).

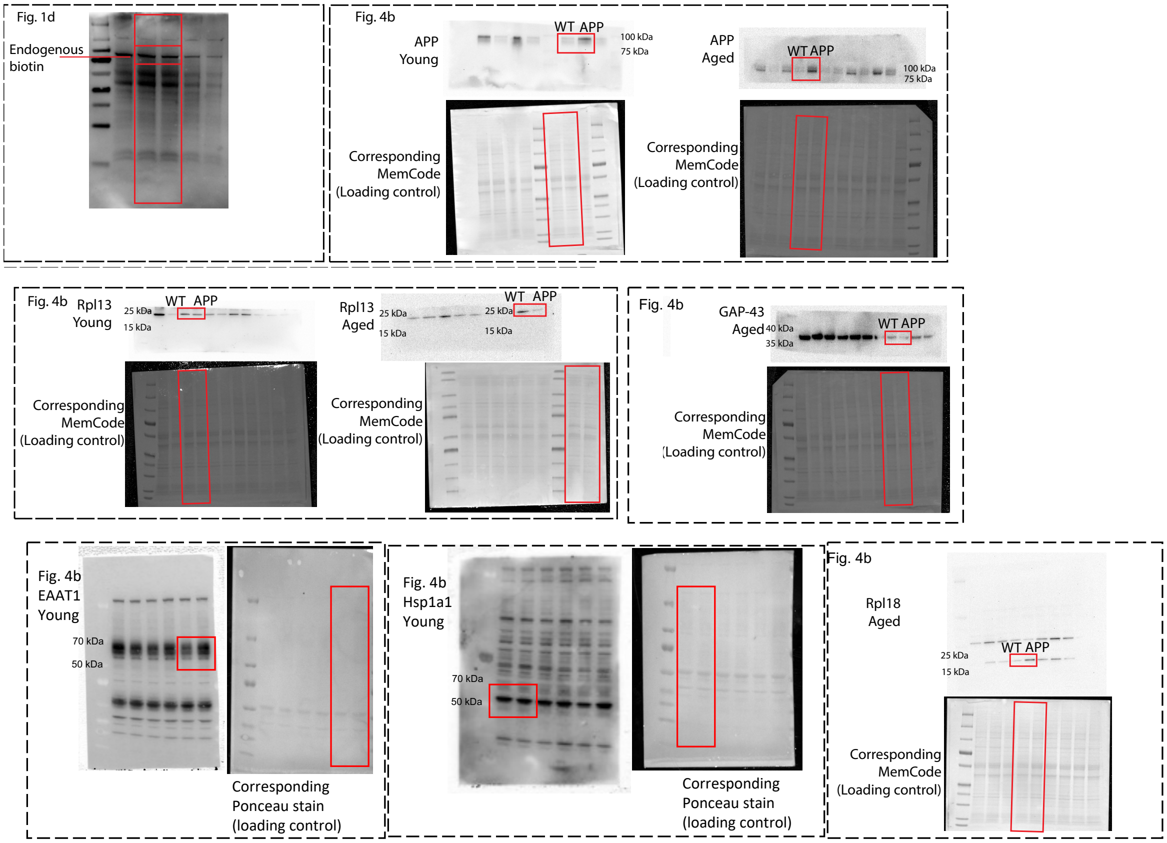


**Supplementary Figure 13. Representative western blots for each antibody used in the manuscript.** Uncropped western blots with corresponding total protein staining (MemCode or Ponceau as indicated) for young and aged APP/PS1 or WT hippocampal lysate samples. Size markers are included for candidate proteins. Whole lanes of total protein stain were analyzed as per the methodological description.

# Supplementary Table

| Cluster 1 | Cluster 2 | | Cluster 3 | | | | | | | | | Cluster 4 | | | | Cluster 5 | | | |
|---|---|---|---|---|---|---|---|---|---|---|---|---|---|---|---|---|---|---|---|
| Cat | Dzank1 | Sumo2 | Rab6a | Khsrp | Psap | Pdha1 | Matr3 | Dnaja2 | Timm13 | Cct7 | Ank2 | Aars | Dstn | Ldha | Rnh1 | Ckmt1 | Olfm1 | Tubb4a | Yars |
| Plek | Sncb | Ube2n | Ppp1cb | Dnajb4 | Camk2b | Sf3b1 | Ctbp1 | Fth1 | Zmiz1 | Ubqln2 | Celf2 | Abi2 | Eef1a2 | Map1lc3b | Rufy3 | Kpnb1 | Grin1 | Psmd4 | Gaa |
| Dpysl5 | Fkbp1a | Prepl | Sept7 | Cadps | Nell2 | Dnajc7 | Cct6a | Zfand5 | Akap12 | U2af2 | Puf60 | Acot7 | Ehd1 | Map2 | Sbds | Fxr1 | Timm10 | Aplp2 | Stx1b |
| Dnajb1 | Ewsr1 | Srcin1 | Spin1 | Idh3a | Cct4 | Ap2a1 | Dhx9 | Stau1 | Hk1 | Kcnab2 | Sparcl1 | Actr1b | Eif4g3 | Map6 | Sgta | Ifrd1 | Ptbp2 | Sirt2 | Camk2d |
| Vcam1 | Tax1bp1 | Dnajc6 | Sf1 | Tcf4 | Hspa9 | Chmp4b | Gatad2b | Rpl36 | Glg1 | Ndufa8 | Ndrg1 | Add1 | Eif4h | Map7d2 | Sik3 | Pabpc1 | Dnaja1 | Cpne6 | Atp5h |
| Atp1b2 | Phactr1 | Uqcrc2 | Camk2a | Mat2a | Tceb2 | Hp1bp3 | Pld3 | Eif4g1 | Elavl4 | Hnrnpd | Sept3 | Ahcyl1 | Eno1 | Mapk1 | Snrpd3 | Hmox1 | Mfge8 | Ndufs8 | Jun |
| Nfasc | Gsta4 | Gmps | Fos | Nsf | Ppp2ca | Hsp90ab1 | Napb | Dctn1 | Maff | Arpc2 | G3bp1 | Ak5 | Eno2 | Mbnl2 | Snx1 | Ctsb | Coro1a | Hspe1 | Syt11 |
| Slc25a12 | Amph | Trim32 | Dlgap1 | Ndufv2 | Sept5 | Tollip | Efhd2 | Atp5a1 | Myo5a | Celf4 | Srsf3 | Aldoa | Fosl2 | Mif | Spock2 | Hnrnpa0 | Rps4x | Npm1 | Clta |
| Ctsd | Arhgef7 | Pcmt1 | Luc7l2 | Trim9 | Sptbn1 | Nptn | Basp1 | Stmn2 | Upf1 | Dpysl3 | Hnrnpa1 | Anp32a | Fscn1 | Mlf2 | Sub1 | Atp6v0d1 | Plp1 | Numbl | Hnrnpab |
| Ctss | Nckap1 | Cplx1 | Dync1h1 | C1qtnf4 | Calu | Gnao1 | Cap1 | Cfl1 | Rtn1 | Atp6v1c1 | Taldo1 | Ap2a2 | Gapdh | Ncdn | Sucla2 | Chp1 | Plat | Erc1 | Clint1 |
| Srsf4 | Soga3 | Mef2d | Edil3 | Gnl3l | Hnrnpk | Clic4 | Ndrg3 | Psma4 | Eef2 | Rbfox1 | Psmc2 | Ap3b2 | Gdi1 | Ndrg4 | Syn1 | Cox6c | Vps35 | Impdh2 | Rbm39 |
| B2m | Ddt | Zcchc18 | Psmd2 | Snrpn | Serbp1 | Ddx5 | Dlg4 | Napg | Txnl1 | Nrcam | | Araf | Glod4 | Nudc | Syn2 | Hspa8 | Acat1 | Cox5b | Dynlrb1 |
| Ap2m1 | Rph3a | Map2k1 | Ctnna2 | G3bp2 | Csde1 | Actr2 | Nedd4l | Pfkm | Esd | Stxbp1 | | Atp6v1a | Gmfb | Pafah1b1 | Synj1 | Timp2 | Fam168a | Rplp2 | Oxr1 |
| Pdia6 | Tpi1 | Arpc1a | Aplp1 | Egr1 | Prkar1b | Hspa4 | Cdkl5 | Nme1 | Prkcg | Aak1 | | Atp6v1b2 | Gpd2 | Pafah1b2 | Tagln3 | Sparc | Ndufa7 | Oat | Lancl1 |
| Hyou1 | Trim28 | Morf4l2 | Psip1 | Nap1l1 | Dctn2 | Clip3 | Rps27a | Akap8l | Gdi2 | Hnrnpu | | Atp6v1d | Gphn | Pak1 | Tbcb | Slc25a22 | Aldh5a1 | Atp1a2 | Hnrnpa2b1 |
| Rpl8 | Phyhip | Irf2bpl | Sfpq | Apbb1 | Ogt | Eif5 | Dhrs1 | Cct5 | Pspc1 | Stmn3 | | Atxn10 | Gpm6a | Pcbp1 | Tcea1 | Slc9a3r1 | Igsf8 | Glul | Gls |
| Aldh2 | Mkl2 | Maged1 | Cct3 | Fis1 | Coro1c | Crym | Rps19 | Eef1a1 | Ppia | Atp6v1e1 | | Atxn2 | Gprasp1 | Pcbp2 | Tnr | Grb2 | Tubb4b | Ncald | Ccsap |
| Rbm3 | Akr1a1 | Icam5 | Lrpap1 | Cyfip2 | Sh3gl2 | Map7d1 | Hnrnph1 | Ndufs1 | Cltc | Ik | | Bsn | Gprasp2 | Pdxk | Tppp | Psmc1 | Adrm1 | Naca | Prdx1 |
| Apoe | Snca | Hook3 | Tceb1 | Hnrnpl | Cntn1 | Lasp1 | Atp5b | Sorbs2 | Tagln2 | Cndp2 | | Camk2g | Hgs | Pfkp | Tpt1 | Ctsl | Hpcal4 | Srsf2 | Dsp |
| Anxa5 | Impact | Cbr1 | Add2 | Hpca | Mapre3 | Capzb | Ywhab | Atp5j | Fubp1 | Mbp | | Cdc37 | Hint1 | Pfn2 | Tufm | Uqcrb | Asrgl1 | Rpl27 | Sri |
| Arglu1 | Abr | Camkv | Tardbp | Smarca4 | Tsc22d1 | Psmc5 | Sugt1 | Atp2b1 | Mdh2 | Chmp2a | | Cend1 | Hmgb1 | Pgk1 | Uba1 | Sdha | Idh3g | Hsp90aa1 | Srsf1 |
| Hsph1 | Qdpr | Bcan | Cnp | Atxn2l | Psma1 | Tpd52l2 | Dlg3 | Atp6v1g2 | Gas7 | Ppp3cb | | Ckap5 | Hnrnph2 | Pkm | Ube2d3 | Psmc6 | Ezr | Safb | Tra2b |
| Adamts4 | Agfg2 | Calb1 | Csnk2b | Clstn1 | Arpc3 | Flnb | Spon1 | Ptk2b | Psmc3 | Baiap2 | | Ckb | Homer1 | Pls3 | Ubqln1 | Tkt | Syncrip | Prkce | Gng2 |
| Pdhb | Prdx5 | Mdh1 | Ap2b1 | Vsnl1 | Ddx17 | Rab5a | Sphkap | Gabarapl2 | Vcp | Stx12 | | Cnbp | Hspa12a | Ppid | Uchl1 | Nptxr | Junb | Synpo | Rps13 |
| Ndufv1 | Gem | Park7 | Sort1 | Epha4 | Prdx6 | Add3 | Son | Elavl3 | Usp9x | Slc25a4 | | Cnn3 | Hspa4l | Ppme1 | Usp5 | Aco2 | Phgdh | Idh1 | Slc3a2 |
| Sarnp | Pgam1 | Psd3 | Ctnna1 | Cpe | Sptan1 | Dnm1 | Map1a | Lmna | Sept7 | Tuba4a | | Cplx2 | Iqsec1 | Ppp2r1a | Wbp2 | Fyttd1 | Rtn3 | Eif2s2 | H1f0 |
| Anxa2 | Nudt3 | Pebp1 | Ctnnb1 | Clstn3 | Pacsin1 | Hspa2 | Prkar1a | Gtf2b | Tspyl4 | Chgb | | Cttn | Khdrbs1 | Ppp3ca | Ywhae | Adamts1 | Rap1b | Hspa5 | |
| Cand1 | Ddx6 | Gad1 | Stip1 | Jup | Hnrnpul2 | Cct8 | Ndufa4 | Snap91 | Bin1 | Cyc1 | | Ddx1 | Khdrbs3 | Prnp | | Atp5o | Rhob | Nufip2 | |
| Stub1 | Gspt1 | Got1 | Nedd4 | Ppp1r2 | Snap47 | Rpsa | Syngap1 | Atp1b1 | Clstn2 | Bcas1 | | Dlg2 | Kif1a | Ptges3 | | Vapa | Syp | Rpl23a | |
| Col1a2 | Usp14 | Ldhb | Scg2 | Nono | Rbm42 | Anxa6 | Cct2 | Ywhah | Caprin1 | Sap18 | | Dmtn | Kif5c | Ptn | | Kif5b | Zeb2 | Sqstm1 | |
| Uqcrc1 | Slc4a10 | Ppp1r10 | Bag6 | Cs | Vim | Snw1 | Snap25 | Gnai1 | Wdr1 | Ociad1 | | Dnm1l | Klc1 | Rbbp7 | | Pa2g4 | Nrgn | Rtn4 | |
| Rpl29 | Clip2 | Cstf2t | Tpm3 | Hist1h4a | Map1b | Rad23b | Cx3cl1 | Dcaf7 | Hnrnpa3 | Eif4a1 | | Dpysl2 | L1cam | Rnf14 | | Ncl | Srsf7 | Syt1 | |
| **PPI enrichment p-value:** | **PPI enrichment p-value:** | | **PPI enrichment p-value:** | | | | | | | | | **PPI enrichment p-value:** | | | | **PPI enrichment p-value:** | | | |
| **6.68E-05** | **6.34E-09** | | **< 1.0e-16** | | | | | | | | | **< 1.0e-16** | | | | **< 1.0e-16** | | | |
| **GO: protein processing in ER** | **GO: synapse** | | **Go: synaptic vesicle cycle** | | | **GO: membrane trafficking** | | | **GO: synapse** | | | **GO: glycolysis and gluconeogenesis** | | | | **GO: myelin sheath** | | **GO: Alzheimer's disease** | |
| **FDR: 3.2E-4** | **FDR: 5.6E-7** | | **FDR: 2.77E-6** | | | **FDR: 6.24E-11** | | | **FDR: 4.82E-29** | | | **FDR: 3.02E-5** | | | | **FDR: 5.17e-18** | | **FDR:8.16e-07** | |

**Supplementary Table 1.** Protein identities contained within key biological pathways as detected by the hierarchical clustering depicted in **Figure 5** and **Supplementary Figures 5-10.** BONLAC-detected proteins observed in both young and aged APP/PS1 and wild-type mouse hippocampus (n = 5-7 mice/genotype/group). PPI and FDR as detected via Cytoscape and StringDb.
