# Supplementary material for: Age-dependent shift in the de novo proteome accompanies pathogenesis in an Alzheimer’s disease mouse model
Source: Commun Biol. 2021 Jun 30;4:823. doi: 10.1038/s42003-021-02324-6 (PMC8245541; doi:10.1038/s42003-021-02324-6)
Supplement: Supplementary file 2 — Description of Additional Supplementary Files [file 42003_2021_2324_MOESM2_ESM.pdf]

Description of Additional Supplementary Files

File name: Supplementary Data 1

Description: We have supplied all data underlying graphs and figures within the manuscript as an Excel file that is described in the following section of this chart.

File name: Supplementary Data 2

Description: We have provided Significance B analyses for each dataset as an Excel file with two sheets:

SignificanceB_rawratios Young: Intensity and normalized H/M ratios for each of the 5 3-5 month old samples included in the analysis, and subsequent Significance B information for this dataset.

SignificanceB_rawratios Aged: Intensity and normalized H/M ratios for each of the 7 12+ month old samples included in the analysis, and subsequent Significance B information for this dataset.
