# Supplementary material for: Age-dependent shift in the de novo proteome accompanies pathogenesis in an Alzheimer’s disease mouse model
Source: Commun Biol. 2021 Jun 30;4:823. doi: 10.1038/s42003-021-02324-6 (PMC8245541; doi:10.1038/s42003-021-02324-6)
Supplement: Supplementary file 5 — Reporting Summary [file 42003_2021_2324_MOESM5_ESM.pdf]

nature research

Corresponding author(s): Eric Klann

Last updated by author(s): May 27, 2021

Reporting Summary

Nature Research wishes to improve the reproducibility of the work that we publish. This form provides structure for consistency and transparency in reporting. For further information on Nature Research policies, see our Editorial Policies and the Editorial Policy Checklist.

Statistics

For all statistical analyses, confirm that the following items are present in the figure legend, table legend, main text, or Methods section.

n/a	Confirmed	
☐	☒	The exact sample size (*n*) for each experimental group/condition, given as a discrete number and unit of measurement
☐	☒	A statement on whether measurements were taken from distinct samples or whether the same sample was measured repeatedly
☐	☒	The statistical test(s) used AND whether they are one- or two-sided<br>*Only common tests should be described solely by name; describe more complex techniques in the Methods section.*
☐	☒	A description of all covariates tested
☐	☒	A description of any assumptions or corrections, such as tests of normality and adjustment for multiple comparisons
☐	☒	A full description of the statistical parameters including central tendency (e.g. means) or other basic estimates (e.g. regression coefficient) AND variation (e.g. standard deviation) or associated estimates of uncertainty (e.g. confidence intervals)
☐	☒	For null hypothesis testing, the test statistic (e.g. *F*, *t*, *r*) with confidence intervals, effect sizes, degrees of freedom and *P* value noted<br>*Give P values as exact values whenever suitable.*
☒	☐	For Bayesian analysis, information on the choice of priors and Markov chain Monte Carlo settings
☒	☐	For hierarchical and complex designs, identification of the appropriate level for tests and full reporting of outcomes
☐	☒	Estimates of effect sizes (e.g. Cohen's *d*, Pearson's *r*), indicating how they were calculated

Our web collection on statistics for biologists contains articles on many of the points above.

Software and code

Policy information about availability of computer code

Data collection	LC-MS was conducted using a Thermo Scientific EASY-nLC 1000 coupled to a Q Exactive, High Field mass spectrometer (ThermoFisher Scientific). Western blots were imaged using FluorchemE (Version 4.1.4).
Data analysis	Raw files obtained from mass spectrometry runs were processed using the MaxQuant computational proteomics platform (Version 1.5.5.179) for peptide identification and quantitation. Fragmentation spectra were searched against the Uniprot mouse protein database (downloaded on 12/20/2017, containing 16,950 non-redundant protein entries, combined with 262 common contaminants). Cluster analysis was performed using Cytoscape (Version 3.7.1), Gene Ontology analysis was performed using the Database for Annotation, Visualization and Integrated Discovery (DAVID, Version 6.8), and biological networks were visualized using String-db (Version 11.0). Western blots were analyzed using ImageStudioLite (Version 5.2.5).

For manuscripts utilizing custom algorithms or software that are central to the research but not yet described in published literature, software must be made available to editors and reviewers. We strongly encourage code deposition in a community repository (e.g. GitHub). See the Nature Research guidelines for submitting code & software for further information.

Data

Policy information about availability of data

All manuscripts must include a data availability statement. This statement should provide the following information, where applicable:

- Accession codes, unique identifiers, or web links for publicly available datasets
- A list of figures that have associated raw data
- A description of any restrictions on data availability

The raw mass spectrometry data generated during this study are available at MassIVE (Center for Computational Mass Spectrometry, University of California, San Diego) with the accession number (ftp://massive.ucsd.edu/MSV000085962/).

Field-specific reporting

Please select the one below that is the best fit for your research. If you are not sure, read the appropriate sections before making your selection.

- [x] Life sciences
- [ ] Behavioural & social sciences
- [ ] Ecological, evolutionary & environmental sciences

For a reference copy of the document with all sections, see nature.com/documents/nr-reporting-summary-flat.pdf

Life sciences study design

All studies must disclose on these points even when the disclosure is negative.

Sample size	Our group has extensive experience in performing BONLAC analysis, which was developed in house. We have employed this method in a variety of systems, including in vitro and ex vivo approaches. Thus, we based our calculations on previous analyses published in this lab, in which a minimum of 3 mass spectrometry runs/condition were used. In this current analysis, we performed 5-7 mass spectrometry runs/condition. Please see the following references:<br>Bowling, H., Bhattacharya, A., Zhang, G., Lebowitz, J. Z., Alam, D., Smith, P. T., Kirshenbaum, K., Neubert, T. A., Vogel, C., Chao, M. V., & Klann, E. (2016). BONLAC: A combinatorial proteomic technique to measure stimulus-induced translational profiles in brain slices. Neuropharmacology, 100, 76–89. https://doi.org/10.1016/j.neuropharm.2015.07.017<br>Bowling, H., Bhattacharya, A., Zhang, G. et al. Altered steady state and activity-dependent de novo protein expression in fragile X syndrome. Nat Commun 10, 1710 (2019). https://doi.org/10.1038/s41467-019-09553-8
Data exclusions	2 samples were excluded from the mass spectrometry analysis of young (4 month-old) mice. The first sample was composed of hippocampal slices from one WT and one APP/PS1 mouse, where the APP/PS1 slices had visibly degraded during the labeling process. The second sample was excluded as the medium-heavy SILAC labeling ratio generated via mass spectrometry as the average incorporation relative to all other samples was 10-fold higher, indicating an abnormality occurred during sample processing.
Replication	BONLAC was carried out with a minimum of five runs per condition, with each run examining hippocampal slices from one WT and one age-matched APP/PS1 animal (n = 5-7 biological replicates made up of 1 APP/PS1 and 1 WT; 5-7 animals of each age per genotype were used in total.<br>A minimum of 5 samples/condition were run for western blot analysis
Randomization	Assignment of SILAC labels to experimental conditions (wild-type or APP/PS1) was randomly alternated between biological replicates to ensure that results were not biased by labelling.
Blinding	Investigators were blinded to animal genotype during data collection and analysis.

Reporting for specific materials, systems and methods

We require information from authors about some types of materials, experimental systems and methods used in many studies. Here, indicate whether each material, system or method listed is relevant to your study. If you are not sure if a list item applies to your research, read the appropriate section before selecting a response.

Materials & experimental systems

n/a	Involved in the study	
☐	☒	Antibodies
☒	☐	Eukaryotic cell lines
☒	☐	Palaeontology and archaeology
☐	☒	Animals and other organisms
☒	☐	Human research participants
☒	☐	Clinical data
☒	☐	Dual use research of concern

Methods

n/a	Involved in the study	
☒	☐	ChIP-seq
☒	☐	Flow cytometry
☒	☐	MRI-based neuroimaging

Antibodies

Antibodies used	The following antibodies were used in this study: For western blot: rabbit anti-APP monoclonal antibody (Abcam, ab32136, clone Y188, lot # GR3248334-7), rabbit anti-EAAT1 polyclonal antibody (Abcam, Ab416, lot # GR3328985-3), rabbit anti-GAP-43 polyclonal antibody (Sigma, ab5220, lot # 3245135), rabbit anti-Hsp1a1 polyclonal antibody (Sigma Aldrich, AV33096, lot # QC2724), anti-Rpl13 polyclonal antibody (Thermofisher, PA5-41715, lot # UD2757834A), and anti-Rpl18 polyclonal antibody (Abcam, ab241988, lot # GR3304969-2). Secondary antibodies were either goat anti-mouse IgG HRP (Promega, W4021, lot # 0000262777) or goat anti-rabbit IgG HRP (Promega, W4011, lot # 0000355714) respectively. For immunohistochemistry: rabbit anti-amyloid beta antibody (Enzo Life Sciences, ENZ-ABS612-0200, clone 6E10, lot # 01172006).
Validation	The anti-APP antibody ab32136 is a knock-out validated antibody as described in the publication Del Turco D, Paul M, Schlaudraff J, Hick M, Endres K, Müller U, et al. Region-Specific Differences in Amyloid Precursor Protein Expression in the Mouse Hippocampus.

Front Mol Neurosci. 2016;9:134. According to Abcam, this antibody is suitable for: WB, IHC-P, Flow Cyt, IP, ICC/IF, and reacts with: Mouse, Rat, Human.
The anti-EAAT1 antibody Ab416 was validated by Abcam using western blot with rat brain cortex. According to Abcam, this antibody is suitable for WB and IHC-P, and reacts with Rat, Mouse and Human.
The anti-GAP-43 antibody ab5220 has been used successfully in the following publication: Kilpatrick, C. L., Murakami, S., Feng, M., Wu, X., Lal, R., Chen, G., Du, K., & Luscher, B. (2016). Dissociation of Golgi-associated DHHC-type Zinc Finger Protein (GODZ)- and Sertoli Cell Gene with a Zinc Finger Domain-β (SERZ-β)-mediated Palmitoylation by Loss of Function Analyses in Knock-out Mice. The Journal of biological chemistry, 291(53), 27371–27386. https://doi.org/10.1074/jbc.M116.732768. According to Abcam, this antibody is suitable for: ELISA, ICC, IHC, IP, WB, and reacts with: Human, Mouse, Rabbit, Chicken, Bovine.
The anti-Hsp1a1 antibody AV33096 was validated by Sigma Aldrich using western blot with fetal liver cells. According to Sigma Aldrich, this antibody is suitable for WB and reacts with goat, guinea pig, mouse, rabbit, sheep, human, dog, bovine, horse, rat.
The anti-Rpl13 antibody PA5-41715 was validated by ThermoFisher Scientific using western blot with Jurkat, HeLa, human fetal liver, HEK293T and MFC7 cells. According to ThermoFisher Scientific, this antibody is suitable for: ICC, IF, IHC and WB, and reacts with: Bovine, Dog, Horse, Guinea pig, Human, Mouse, Rabbit, Rat, Zebrafish.
The anti-Rpl18 antibody ab241988 was validated by Abcam using western blot with HeLa, HEK-293T, Jurkat, TCMK-1 and NIH/3T3 cells. According to Abcam, this antibody is suitable for WB, and reacts with: Mouse, Human.

Animals and other organisms

Policy information about studies involving animals; ARRIVE guidelines recommended for reporting animal research

Laboratory animals	This study used APP/PS1 transgenic mice (B6;C3-Tg(APPswe, PSEN1/dE9)85Dbo/Mmjax) and wild-type littermates, bred and maintained on C57-BL6 and B6.C3 (Jackson Labs) backgrounds. Two age groups were used: 3-5 months of age, and >12 months of age. Mice of both sexes were used.
Wild animals	The study did not involve wild animals.
Field-collected samples	The study did not involve samples collected from the field.
Ethics oversight	This study protocol was reviewed and approved by the New York University Animal Welfare Committee and followed the National Institutes of Health (NIH) Guide for the Care and Use of Laboratory Animals

Note that full information on the approval of the study protocol must also be provided in the manuscript.
